# Indicators of emotional distress and mindfulness in undergraduate students: a cross-sectional study

**DOI:** 10.1590/0034-7167-2023-0499

**Published:** 2024-10-07

**Authors:** Vanessa Ferraz Leite, Moisés Kogien, Margani Cadore Weis Maia, Marina Nolli Bittencourt, Larissa de Almeida Rézio, Samira Reschetti Marcon

**Affiliations:** I Universidade Federal de Mato Grosso. Cuiabá, Mato Grosso, Brazil

**Keywords:** Students, Universities, Mental Health, Emotional Distress, Mindfulness, Estudiantes, Universidades, Salud Mental, Estrés Emocional, Atención Plena

## Abstract

**Objectives:**

to assess the association between indicators of emotional distress and dispositional mindfulness in health students at a Brazilian federal public university.

**Methods:**

a cross-sectional study, developed with university students in the health area of a public institution from May to June 2022. In the analysis, multiple linear regression was used using SPSS software.

**Results:**

the sample was mostly female, ≤ 22 years old, non-white, studying the first semesters, with a higher prevalence for the medicine course. Students presented moderate dispositional mindfulness scores. It was observed that the variables of stress, depression and current suicide risk were associated with the capacity for mindfulness.

**Conclusions:**

knowing the indicators of emotional distress that are related to the potential of mindfulness can contribute as a situational diagnosis to better design strategies that promote the improvement of emotional indicators of health students.

## RESUMEN

**Objetivos:**

evaluar la asociación entre indicadores de malestar emocional y mindfulness disposicional en estudiantes de salud de una universidad pública federal brasilena.

**Métodos:**

estudio transversal, desarrollado con estudiantes universitarios del área de salud de una institución pública de mayo a junio de 2022. En el análisis se utilizo regresión lineal múltiple mediante el software SPSS.

**Resultados:**

la muestra fue mayoritariamente femenina, ≤ 22 anos, no blanca, cursando los primeros semestres, con mayor prevalencia para la carrera de medicina. Los estudiantes presentaron puntuaciones moderadas en mindfulness disposicional y se observo que las variables estrés, depresión y riesgo de suicidio actual se asociaron con la capacidad de mindfulness.

**Conclusiones:**

conocer los indicadores de sufrimiento emocional que se relacionan con el potencial del mindfulness puede contribuir como diagnóstico situacional a disenar mejores estrategias que promuevan la mejora de los indicadores emocionales de los estudiantes de salud.

## INTRODUCTION

In the health field, the term “mindfulness” refers to the mental state of consciously and deliberately paying attention to the experience of the here and now, with curiosity, monitoring, openness, acceptance and without judgment^(1, 2)^. In this way, scientific studies understand the capacity for dispositional mindfulness (DM) as a construct with central characteristics of maintaining the ability to be attentive and aware of the present moment in everyday life^(3)^, and its relevance lies in maintaining capabilities that interact with different cognitive, emotional and motivational aspects for personal and social development^(4, 5)^. Practical or experiential in nature, DM helps individuals with knowledge about their own thought processes, i.e., with metacognition. It is known that metacognition, mindfulness and non-judgmental awareness of the present are therefore related to mindfulness cultivation, leading to greater awareness and understanding of one’s own experiences, thoughts, emotions and bodily sensations^(1, 2)^.

At the university level, the different cognitive activities to which students are exposed require concentration and the effectiveness of mindfulness to facilitate learning and have good academic per-formance^(6)^. However, certain factors, in association, can harm the balance of students’ attentional and emotional self-regulation^(7, 8)^. With regard to health students, demands such as the requirement for academic excellence, the need to acquire clinical skills for care^(9)^, the development of interpersonal skills to deal with the suffering of others and the competence to solve problems are directly linked to attentional skills and emotional regulation^(10)^. In this context, mindfulness, which refers to the ability to remain intentionally attentive and mentally present without wandering^(2)^, becomes an important ally for students as it enables the process of assimilation and emotional coping in the midst of transience of events^(11)^.

Scientifically, DM correlates with several indicators of physical and mental health, such as maintaining the balance of the sympathetic and parasympathetic autonomic nervous system, high levels of positive affect, contentment with life, vitality, negative affects and symptoms of mental distress^(1)^. Although DM cultivation is an important element in the university environment as it promotes learning, some indicators of emotional distress (IED) can interfere with the way students use this mindfulness, thus compromising its effectiveness.

IED refer to a set of variables that allow identifying continuous negative reactions that prevent the flourishing of human potential^(12, 13)^. Among them, there are symptoms of depression, anxiety, stress, suicidal ideas and daytime drowsiness, and scientific studies show an increase in these emotional indicators in undergraduate students^(14, 15)^.

The rise of some of these IED and their associations with mindfulness significantly affect academic activities as there is a decline in DM capacity^(11, 16)^. Thus, despite individuals showing a considerable level of attention, due to numerous demands or circumstances, which are not always well assimilated, they end up developing symptoms of depression and anxiety, which can result, for instance, in academic difficulties. However, DM can be improved and cultivated through training programs and interventions^(2)^. A study with medical students demonstrated that DM encouragement contributed to reducing exhaustion and promoted well-being, and could, therefore, be incorporated through training and inclusion in undergraduate health programs^(17)^, improving students’ DM capacity and, therefore, its potential salutary effects on physical and mental health.

Considering this overview, this study hypothesizes that IED are negative predictors undergraduate health students’ DM capacity, i.e., the higher the level of emotional distress of these students, the lower the DM capacity measured will be.

## OBJECTIVES

To assess the association between IED and DM in health students at a Brazilian federal public university.

## METHODS

### Ethical aspects

The research was approved by the Research Ethics Committee and has a Certificate of Presentation of Ethical Appreciation.

### Study design, period and place

This is a cross-sectional study guided by STrengthening the Reporting of OBservational studies in Epidemiology (STROBE)^(18)^.

The study was carried out from May to June 2022 at a federal public university in the Brazilian midwestern.

### Population and sample; inclusion and exclusion criteria

The target population was made up of 833 students from health courses (nursing, medicine, nutrition and psychology), regularly enrolled on the study site’s main campus. All students who were in the classroom on collection days were invited to participate in the study, considering the inclusion criterion to be 18 years of age or older. Students on internship were not part of the study, as they were not on *campus* during the collection period. The sample was selected for convenience, based on students’ availability and acceptance to participate in the study. A total of 617 students were recruited, six of whom were excluded due to incomplete questionnaires. Therefore, the final sample consisted of 611 participants (73.35% of the target population).

### Study protocol

Data were collected using a closed-ended, self-completion instrument, composed of sociodemographic (sex, age, self-declared skin color, religious practice, sexual orientation, marital, work and housing situation) and academic variables (school semester and course enrolled). The instrument was developed based on studies by the Brazilian National Forum of Deans of Community and Student Affairs^(19)^.

The Mindful Attention Awareness Scale (MAAS)^(20)^, validated in Brazil^(3)^, was used to assess the level of DM. It consists of 15 sentences about the level of mindfulness in everyday situations, with answers on a six-point Likert scale, ranging from 1 - almost always to 6 - almost never and a total score of 15 and 90 points. In this study, internal consistency was 0.86 (McDonald’s ω).

To assess symptoms of anxiety, depression and stress, the Depression, Anxiety, and Stress Scale (DASS-21)^(21)^, validated in Brazil^(22)^, was applied for use in the university population^(23)^. It has 21 items with responses on a four-point Likert scale, divided into three subscales, each with seven items. The higher the total score for each subscale, the greater the symptoms of anxiety, depression and stress. In this study, internal consistency for depression, anxiety and stress subscales was, respectively, 0.94, 0.89 and 0.99 (McDonald’s ω).

During collection, the Mini International Neuropsychiatric Interview (MINI) module C was also used to assess the risk of suicide in the last month. It has six items with dichotomous answers (yes or no). Provides an overall score from 0 to 33 points. In this study, participants were stratified as follows: score 0 (no risk of suicide); scores between 1 and 33 points (some current suicide risk). This is an instrument translated and validated for the Brazilian context with good psychometric performance^(24)^.

Finally, the degree of daytime sleepiness was assessed using the Epworth Sleepiness Scale, an instrument translated and validated for the Brazilian context^(25)^. It has eight items with responses on a three-point Likert scale (0 - no probability of falling asleep to 3 - strong probability of falling asleep). In this study, internal consistency was 0.99 (McDonald’s ω).

Before applying the instruments to collect data, a pilot test was carried out in April 2022, with 56 students from the first three semesters of an exact sciences course at the same participating institution. In the pilot test, good applicability of instruments was demonstrated as well as a good average time to complete (45 minutes).

Data collection took place in person with classroom visits on days defined in a prior schedule. After authorization from the professor in the classroom, a brief explanation of the research was carried out and the Informed Consent Form (ICF) was signed. The ICF was obtained from all individuals involved in the study in writing. Participants responded to self-completion instruments individually and, at the end, deposited them in a sealed urn placed in front of the room, aiming to guarantee response anonymity security. Data were collected by the main researcher and eight duly trained academics from nursing, medicine, psychology and public health courses.

### Data analysis, and statistics

In the analysis, continuous variables were expressed as mean and standard deviation, whereas categorical variables were presented as absolute and relative frequencies. To carry out comparative analyzes between the mean scores of DM and IED, t-tests were used for independent groups, with magnitude of effect estimated using Cohen’s d test. Moreover, 95% Confidence Intervals were obtained using the bootstrapping technique with 1,000 resamples, which provides bias-corrected and accelerated confidence intervals (BCaCI). This technique was used to correct deviations from the normality of sampling distribution and differences between the sizes of compared groups.

Furthermore, multiple analysis of associated factors was carried out using the multiple linear regression technique. To construct the final model adopted, all explanatory variables related to emotional distress were included in the model and adjusted for the sociodemographic and academic characteristics used in this study. Before assuming the final model, multiple linear regression assumptions were checked, including the normality of the distribution of residuals, checking the absence of multicollinearity using the Variance Inflation Factor (VIF) less than ten and confirming non-occurrence of residual autocorrelation (Durbin-Watson =1.882). All analyzes were performed using the Statistical Package for the Social Sciences (SPSS) version 27.

## RESULTS

The sample of students was characterized as predominantly female (73.3%), with a median age ≤ 22 years. The largest proportion of students declared themselves to have non-white skin color (57.1%), heterosexual orientation (71.5%), without a marital partner (67.4%) and not living alone during their academic training (81.5%). Furthermore, most students reported carrying out some religious practice (58.6%) and just dedicating themselves to studies, without the need to combine work activities (77.9%) (Table 1).

**Table 1:** Sociodemographic and academic profile of health students at a public university in midwestern Brazil (N = 611), Cuiabá, Mato Grosso, Brazil, 2022

Sociodemographic/academic characteristics	n (%)
Sex	
Female	448 (73.3%)
Male	161 (26.4%)
Not reported	02 (0.3%)
Median age	
≤ 22 years	384 (62.8%)
> 23 years	227 (36.3%)
Self-declared skin color	
Non-white	349 (57.1%)
White	262 (42.9%)
Sexual orientation	
Heterosexual	437 (71.5%)
Minority sexual orientations	168 (27.5%)
Not reported	06 (1.0%)
Religious practice	
Yes	358 (58.6%)
No	252 (41.2%)
Not reported	01 (0.2%)
*Marital status*	
With a partner	194 (31.8%)
Without a partner	412 (67.4%)
Not reported	05 (0.8%)
Housing situation	
Lives alone	110 (18.0%)
Does not live alone	498 (81.5%)
Not reported	03 (0.5%)
Employment situation	
Works and studies	128 (21.0%)
Only studies	476 (77.9%)
Not reported	07 (1.1%)
Course period	
1^st^ or 2^nd^ semester	163 (26.7%)
3^rd^ or 4^th^ semester	194 (31.8%)
5^th^ or 6^th^ semester	143 (23.4%)
7^th^ or 8^th^ semester	107 (17.5%)
Not reported	04 (0.7%)
Course	
Nursing	136 (22.3%)
Nutrition	114 (18.7%)
Psychology	176 (28.8%)
Medicine	185 (30.3%)

In relation to the academic profile, students from the 1^st^ to 8^th^ semester participated, with a greater participation of students from the 3^rd^ or 4^th^ semesters (31.8%). Medical students predominated in the final sample analyzed (30.3%).

The total sample presented moderate levels of DM (**x̅** = 51.75; standard deviation = 12.67). Analysis by subgroups created from the IED demonstrated differences between students with evidence of suffering, when compared to those without (Table 2).

**Table 2:** Relationship between indicators of emotional distress and dispositional mindfulness in health students, Cuiabá, Mato Grosso, Brazil,2022

**Indicators of emotional distress**	**Frequency n (%)**	**Mean (standard deviation)**	**Dispositional mindfulness**		**Cohen’s d**
			**t**	***p* value (BCa 95%CI)**	
Depression					
No symptoms	287 (47.0%)	57.08 (12.00)	10.872	<0.001 (8.61; 12.04)	0.88
With symptoms	319 (52.2%)	46.84 (11.18)			
Did not answer**	05 (0.8%)				
Anxiety					
No symptoms	158 (25.9%)	56.95 (12.41)	6.273	<0.001 (4.89; 9.70)	0.58
With symptoms	448 (73.3%)	49.83 (12.21)			
Did not answer**	05 (0.8%)				
Stress					
No symptoms	373 (61.0%)	56.02 (11.54)	11.689	<0.001 (9.30; 12.98)	0.97
With symptoms	236 (38.7%)	44.90 (11.27)			
Did not answer**	02 (0.3%)				
Current suicide risk*					
Without risk	352 (57.6%)	54.70 (13.01)	7.040	<0.001 (5.22; 9.01)	0.58
With some risk	258 (42.2%)	47.67 (10.98)			
Did not answer**	01 (0.2%)				
Excessive daytime sleepiness					
Normal	266 (43.5%)	53.91 (12.78)	3.670	<0.001 (1.91; 5.66)	0.30
Excessive daytime sleepiness	339 (55.5%)	50.13 (12.35)			
Did not answer**	06 (1.0%)				

*Equal variances not assumed by Levene’s test in the comparative analysis for means of the DM variable; **Due to the small sample size of individuals who did not respond to the emotional distress assessment instruments, the comparison between groups was only between the groups with emotional distress and without distress.

It was identified that 52.2% of students presented clinically significant depressive symptoms; 73.3% had symptoms of anxiety; 38.7% presented symptoms compatible with stress; 42.2% had some current risk of suicide; and 55.5% had symptoms of excessive daytime sleepiness.

Multiple linear regression analysis demonstrated that, among the indicators of mental distress analyzed, stress (p <0.001; BCa 95%CI = −9.647; −5.182), depression (p<0.001; BCa 95%CI = −7.706; −2.828) and current suicide risk (p = 0.006; BCa 95% CI = −4.517; −0.893) were negatively and significantly associated with DM, with stress having greater explanatory competence (P = −0.286, p < 0.001). It is noteworthy that the final model assumed was statistically significant (F = 17.684, p < 0.001, R^2^ = 0.267), indicating that the set of variables retained managed to explain 26.7% of the outcome (Table 3).

**Table 3:** Multiple linear regression model for factors associated with dispositional mindfulness in a sample of undergraduate health students, Cuiabá, Mato Grosso, Brazil, 2022

Variables	B	Standard error	β	BCa 95%CI		*p* value	VIF
				Minimum	Maximum		
Intercept	48.161	6.7890		34.805	61.517	< 0.001	
Depression	−5.257	1.120	−0.207	−7.706	−2.828	< 0.001	1.552
Anxiety	−1.063	1.209	−0.037	−3.405	1.204	0.385	1.347
Stress	−7.445	1.105	−0.286	−9.647	−5.182	< 0.001	1.418
Current suicide risk	−2.722	0.922	-0.106	−4.517	−0.893	0.006	1.297
Excessive daytime sleepiness	−1.790	0.944	−0.070	−3.714	0.096	0.062	1.058

*Insertion method: ENTER; R2: 0.267; F: 17.184 (p < 0.001); Durbin Watson: 1,882. Effects of emotional health indicators controlled by sociodemographic and academic variables considered in this study; VIF- Variance Inflation Factor; CI – Confidence Interval.*

## DISCUSSION

This research identified moderate DM scores and obtained the IED depression, stress and suicide risk associated with DM levels in a sample of nursing, medicine, nutrition and psychology students. Moderate DM scores are related to those obtained in research carried out with similar populations^(26, 27, 8)^.

In relation to the prevalence of IED described here, research reaffirms the vulnerability of this population due to transition to university and the numerous adaptive processes faced with or without success^(29, 30)^. Research with 424, 128 Brazilian undergraduate students, in 2018, showed an increase in emotional distress in this population^(19)^, which worsened with the long pandemic period and its added complexity^(29, 30)^.

Therefore, this research is in line with the literature by showing that more than half of health students, after a recent period of pandemic isolation, presented depressive symptoms, symptoms of excessive daytime sleepiness and a high rate of anxiety symptoms. Prevalence obtained is worrying, as such indicators, when related to DM, predispose to weak self-regulation skills, greater vulnerability to mind wandering, damage to cognition, rumination of negative emotions and emotional feelings, inhibition of eudemonic/positive emotions, precariousness of bodily consciousness (interoceptive and proprioceptive abilities), constant motivational dysfunctions, among others^(7, 31, 32, 33, 34)^. These conditions can directly interfere with academic performance as well as these students’ training process^(9, 33, 35)^.

DM comprises the ability to self-regulate attention, open orientation to one’s own lived experience^(1, 2)^, ability to perceive bodily sensations or be aware of one’s own body (interoception and proprioception) and change one’s perspective^(36)^. Regarding emotional experiences, even though they are altered by social and cultural influences, their strongest nature is innate and instinctive^(12)^. This fact means that the ability to self-regulate is variable, and this directly depends on the ability to regulate voluntary attention^(37)^. Thus, despite being a question of an evolutionary and genetic level, DM can be cultivated and expanded uniquely^(2)^, and can protect young people from excessive worries, rumination and fear^(38)^.

The associations obtained in this study confirm that increasing IED scores reduces DM in line with findings from previous literature. Research shows that difficulty responding to stress, symptoms of depression and the presence of suicidal ideation have a negative influence on DM in health students^(11, 16)^.

Degrees in the health area differ from other academic training, due to their high potential for mental illness, mainly due to the interactive nature with human suffering in moments of physical and mental fragility that such courses require. Insecurities and worries can increase students’ stress levels, negatively impacting their mental and emotional health and ability to concentrate^(8, 10, 11)^.

Thus, a Brazilian study carried out with undergraduate nursing students showed a considerable decrease in DM due to insufficient hours of sleep and the presence of stress symptoms^(26)^.

Stress, despite not being an emotion, is an emotional experience associated with biochemical, physiological, cognitive and behavioral aspects^(23)^, or even multiple dimensions, which include the internal (psychophysiology) and the external (environment), in addition to material, social and cultural interactions experienced in a given everyday context^(2)^. In the context of contemporary life, including health students’ university experience and its particularities, the response to persistent stress greatly overloads physiological, humoral and neural resources^(8, 16)^, which can lead to a decrease in DM. Among health students and professionals, the reflection of decreased DM levels generates, among others, neglect of personal care, lack of self-compassionate attitude, difficulties in relating in groups and being compassionate towards others^(39, 40, 41)^.

Another IED associated with a decrease in DM was depressive symptoms. It is known about the complex interaction of depression between social, psychological and biological factors^(42)^. Life events, such as adversities, losses, among others, contribute and can catalyze its development^(42, 43)^. As an important predictor, depressive symptoms, especially among young university students faced with adversity, can signal various health problems, especially in cognitive, behavioral and relational functions strictly linked to the ability to have DM, directly affecting academic performance and other spheres of life^(10)^.

The last association obtained was current suicide risk, which contributed to decreased DM capacity. The current suicide risk is understood not as an emotion, but rather as an evaluative form of deep emotional distress that considers an entire investigative process of detected suicidal behavior^(43)^. A study carried out with Chinese medical students showed that suffering individuals who are at risk of suicide may have difficulty dealing with emotions and have low emotional regulation, which, in turn, can lead to an acute or chronic decrease in skills such as to DM^(10)^.

Therefore, DM cultivation through using bodily-mental strategies based on mindfulness or associated with self-compassionate approaches can motivate experiences of awareness and acceptance of the present moment, and can help individuals to identify their emotional distress and be less burdened by predominant symptoms, such as anxiety, despair and depression^(2, 39, 44)^, which emerge in moments of high stress load similar to those recently experienced with the global COVID-19 crisis^(40)^.

Recently, research has shown that mindfulness programs associated with other practices such as yoga significantly improved the level of mindfulness, self-compassion and compassion of nursing students^(41)^ as well as exerting a protective influence on undergraduate students’ well-being^(45)^.

Furthermore, it is important to highlight that, although the emotional indicator of anxiety was not significant in this study, recent research shows that health students may have a reduction in DM when they present symptoms of anxiety (apprehension, worry and fear), which are generally accompanied by physical symptoms, such as muscle tension, sleep disturbances, restlessness, rapid breathing, tremors and fatigue, which can cause damage to personal and collective life both inside and outside the academic environment^(11, 40, 41)^.

### Study limitations

Given the findings, it is believed that this study makes an important scientific contribution; however, it presents some limitations to be described, such as the type of cross-sectional design, which measures variables simultaneously, without structural distinction between predictor and outcome variables, allowing restricted conclusions about causality or temporal precedence. It should also be noted that the convenience sample and the fact that the study was carried out on just one of the *campuses* of a single public higher education institution may not reflect the entire population’s representative characteristics. In the future, new studies may cover a broader sample base and include health students from different institutions, regions, states or countries.

### Contributions to nursing

The findings can expand the scope of knowledge, aiming to improve health care management among undergraduate health students. The research can be useful for planning university policies and promoting health students’ mental health, considering the healthy potential of DM in people’s lives.

## CONCLUSIONS

The sample of health students in this study showed moderate DM scores and that increased IED of stress, depression and current suicide risk were associated with decreased DM in the group investigated. In this regard, the situational diagnosis promoted by this research can contribute to developing more assertive strategies regarding emotional regulation and DM strengthening promotion among students studying health courses.

## Supplementary Material

0034-7167-reben-77-05-e20230499-suppl01

## Data Availability

https://doi.org/10.48331/scielodata.GPEFJP
