# Supplementary material for: Indicators of emotional distress and mindfulness in undergraduate students: a cross-sectional study
Source: Rev Bras Enferm. 2024 Oct 7;77(5):e20230499. doi: 10.1590/0034-7167-2023-0499 (PMC11458143; doi:10.1590/0034-7167-2023-0499)
Supplement: 0034-7167-reben-77-05-e20230499-suppl01 [file 0034-7167-reben-77-05-e20230499-suppl01.pdf]

CURSO	SEXO	COR_PELE	PRATICA_FE	ORIENT_SEXUAL	STATUS_CONJUGAL	TRABALHO	MORADIA	SEMESTRE_MATRICULADO	MAAS_TOTAL	RISCODESUICIDIO_ESCORE	ESCORE_DEPRESSAO	ESCORE_ANSIEDADE	ESCORE_ESTRESSE	TOTAL_EPWORTH
NUTRICAO	FEMININO	BRANCA	NÃO	MINORIAS SEXUAIS	COM COMPANHEIRO	SÓ ESTUDA	NÃO MORA SÓ	0.00	49.00	3.00	18.00	19.00	25.00	10.00
NUTRICAO	MASCULINO	BRANCA	NÃO	HETEROSSEXUAL	SEM COMPANHEIRO	SÓ ESTUDA	NÃO MORA SÓ	0.00	51.00	4.00	18.00	17.00	15.00	16.00
NUTRICAO	FEMININO	NÃO BRANCA	SIM	HETEROSSEXUAL	SEM COMPANHEIRO	SÓ ESTUDA	NÃO MORA SÓ	0.00	64.00	0.00	22.00	21.00	22.00	15.00
NUTRICAO	FEMININO	NÃO BRANCA	SIM	MINORIAS SEXUAIS	SEM COMPANHEIRO	SÓ ESTUDA	NÃO MORA SÓ	0.00	53.00	9.00	17.00	20.00	21.00	15.00
NUTRICAO	MASCULINO	NÃO BRANCA	SIM	HETEROSSEXUAL	SEM COMPANHEIRO	SÓ ESTUDA	NÃO MORA SÓ	0.00	55.00	0.00	11.00	20.00	19.00	
NUTRICAO	FEMININO	NÃO BRANCA	SIM	HETEROSSEXUAL	COM COMPANHEIRO	SÓ ESTUDA	NÃO MORA SÓ	0.00	61.00	0.00	18.00	14.00	18.00	13.00
NUTRICAO	FEMININO	NÃO BRANCA	NÃO	HETEROSSEXUAL	SEM COMPANHEIRO	SÓ ESTUDA	NÃO MORA SÓ	0.00	61.00	0.00	8.00	10.00	13.00	2.00
NUTRICAO	FEMININO	NÃO BRANCA	SIM	HETEROSSEXUAL	SEM COMPANHEIRO	ESTUDA E TRABALHA	NÃO MORA SÓ	0.00	43.00	0.00	15.00	8.00	13.00	11.00
NUTRICAO	FEMININO	NÃO BRANCA	SIM	HETEROSSEXUAL	SEM COMPANHEIRO	ESTUDA E TRABALHA	NÃO MORA SÓ	0.00	46.00	0.00	13.00	14.00	17.00	13.00
NUTRICAO	FEMININO	NÃO BRANCA	NÃO	MINORIAS SEXUAIS	COM COMPANHEIRO	SÓ ESTUDA	MORA SÓ	0.00	57.00	7.00	20.00	18.00	16.00	8.00
NUTRICAO	FEMININO	NÃO BRANCA	SIM	HETEROSSEXUAL	SEM COMPANHEIRO	SÓ ESTUDA	NÃO MORA SÓ	0.00	46.00	1.00	12.00	12.00	19.00	13.00
NUTRICAO	FEMININO	BRANCA	SIM	HETEROSSEXUAL	SEM COMPANHEIRO	ESTUDA E TRABALHA	NÃO MORA SÓ	0.00	62.00	0.00	10.00	13.00	16.00	8.00
NUTRICAO	FEMININO	BRANCA	NÃO	HETEROSSEXUAL	SEM COMPANHEIRO	ESTUDA E TRABALHA	NÃO MORA SÓ	0.00	45.00	0.00	20.00	24.00	24.00	16.00
NUTRICAO	FEMININO	BRANCA	SIM	HETEROSSEXUAL	SEM COMPANHEIRO	SÓ ESTUDA	NÃO MORA SÓ	0.00	43.00	6.00	22.00	19.00	19.00	5.00
NUTRICAO	FEMININO	BRANCA	SIM	HETEROSSEXUAL	SEM COMPANHEIRO	SÓ ESTUDA	MORA SÓ	0.00	16.00	0.00	8.00	7.00	9.00	10.00
NUTRICAO	FEMININO	NÃO BRANCA	NÃO	HETEROSSEXUAL	SEM COMPANHEIRO	SÓ ESTUDA	NÃO MORA SÓ	0.00	30.00	0.00	20.00	24.00	21.00	8.00
NUTRICAO	FEMININO	NÃO BRANCA	SIM	HETEROSSEXUAL	COM COMPANHEIRO	SÓ ESTUDA	NÃO MORA SÓ	0.00	42.00	0.00	8.00	13.00	19.00	8.00
NUTRICAO	FEMININO	NÃO BRANCA	SIM	HETEROSSEXUAL	SEM COMPANHEIRO	SÓ ESTUDA	NÃO MORA SÓ	0.00	47.00	0.00	21.00	18.00	19.00	17.00
NUTRICAO	FEMININO	BRANCA	NÃO	HETEROSSEXUAL	SEM COMPANHEIRO	SÓ ESTUDA	NÃO MORA SÓ	0.00	42.00	0.00	14.00	9.00	18.00	8.00
NUTRICAO	FEMININO	NÃO BRANCA	SIM	HETEROSSEXUAL	SEM COMPANHEIRO	SÓ ESTUDA	NÃO MORA SÓ	0.00	42.00	0.00	13.00	11.00	15.00	16.00

NUTRICAO	FEMININO	NÃO BRANCA	NÃO	HETEROSSEXUAL	SEM COMPANHEIRO	ESTUDA E TRABALHA	MORA SÓ	0.00	37.00	0.00	12.00	9.00	14.00	18.00
NUTRICAO	FEMININO	NÃO BRANCA	SIM	HETEROSSEXUAL	COM COMPANHEIRO	SÓ ESTUDA	NÃO MORA SÓ	0.00	34.00	9.00	25.00	27.00	26.00	19.00
NUTRICAO	FEMININO	BRANCA	SIM	HETEROSSEXUAL	SEM COMPANHEIRO	SÓ ESTUDA	NÃO MORA SÓ	0.00	34.00	19.00	26.00	23.00	25.00	21.00
NUTRICAO	FEMININO	NÃO BRANCA	NÃO	MINORIAS SEXUAIS	SEM COMPANHEIRO	SÓ ESTUDA	NÃO MORA SÓ	0.00	70.00	4.00	9.00	12.00	15.00	10.00
NUTRICAO	FEMININO	NÃO BRANCA	SIM	HETEROSSEXUAL	COM COMPANHEIRO	SÓ ESTUDA	NÃO MORA SÓ	0.00	40.00	4.00	21.00	15.00	21.00	15.00
NUTRICAO	FEMININO	NÃO BRANCA	SIM	HETEROSSEXUAL	SEM COMPANHEIRO	SÓ ESTUDA	NÃO MORA SÓ	0.00	37.00	9.00	21.00	18.00	18.00	13.00
NUTRICAO	FEMININO	BRANCA	SIM	MINORIAS SEXUAIS	SEM COMPANHEIRO	SÓ ESTUDA	NÃO MORA SÓ	0.00	43.00	13.00	14.00	19.00	21.00	3.00
NUTRICAO	FEMININO	NÃO BRANCA	NÃO	HETEROSSEXUAL	SEM COMPANHEIRO	SÓ ESTUDA	NÃO MORA SÓ	1.00	59.00	5.00	19.00	19.00	24.00	9.00
NUTRICAO	FEMININO	NÃO BRANCA	NÃO	HETEROSSEXUAL	COM COMPANHEIRO	SÓ ESTUDA	NÃO MORA SÓ	1.00	51.00	33.00	10.00	17.00	19.00	6.00
NUTRICAO	FEMININO	NÃO BRANCA	SIM	HETEROSSEXUAL	SEM COMPANHEIRO	ESTUDA E TRABALHA	NÃO MORA SÓ	1.00	48.00	0.00	11.00	14.00	20.00	18.00
NUTRICAO	FEMININO	NÃO BRANCA	SIM	HETEROSSEXUAL	COM COMPANHEIRO	SÓ ESTUDA	NÃO MORA SÓ	1.00	82.00	0.00	7.00	12.00	13.00	8.00
NUTRICAO	MASCULINO	BRANCA	SIM	HETEROSSEXUAL	SEM COMPANHEIRO	SÓ ESTUDA	NÃO MORA SÓ	1.00	64.00	0.00	10.00	9.00	12.00	7.00
NUTRICAO	FEMININO	NÃO BRANCA	SIM	HETEROSSEXUAL	COM COMPANHEIRO	SÓ ESTUDA	NÃO MORA SÓ	1.00	73.00	0.00	10.00	8.00	12.00	12.00
NUTRICAO	FEMININO	NÃO BRANCA	SIM	HETEROSSEXUAL	COM COMPANHEIRO	SÓ ESTUDA	NÃO MORA SÓ	1.00	77.00	0.00	10.00	11.00	21.00	11.00
NUTRICAO	FEMININO	BRANCA	NÃO	MINORIAS SEXUAIS	SEM COMPANHEIRO	ESTUDA E TRABALHA	NÃO MORA SÓ	1.00	35.00	0.00	18.00	17.00	21.00	11.00
NUTRICAO	FEMININO	BRANCA	NÃO	HETEROSSEXUAL	COM COMPANHEIRO	SÓ ESTUDA	NÃO MORA SÓ	1.00	45.00	0.00	11.00	8.00	14.00	12.00
NUTRICAO	FEMININO	NÃO BRANCA	SIM	HETEROSSEXUAL	COM COMPANHEIRO	ESTUDA E TRABALHA	NÃO MORA SÓ	1.00	44.00	19.00	21.00	19.00	24.00	11.00
NUTRICAO	FEMININO	NÃO BRANCA	SIM	HETEROSSEXUAL	SEM COMPANHEIRO	ESTUDA E TRABALHA	NÃO MORA SÓ	1.00	45.00	0.00	11.00	12.00	11.00	13.00
NUTRICAO	FEMININO	NÃO BRANCA	SIM	HETEROSSEXUAL	SEM COMPANHEIRO	SÓ ESTUDA	NÃO MORA SÓ	1.00	45.00	2.00	13.00	17.00	21.00	19.00
NUTRICAO	FEMININO	NÃO BRANCA	SIM	HETEROSSEXUAL	COM COMPANHEIRO	SÓ ESTUDA	NÃO MORA SÓ	1.00	60.00	1.00	15.00	9.00	13.00	7.00
NUTRICAO	FEMININO	NÃO BRANCA	SIM	HETEROSSEXUAL	SEM COMPANHEIRO	SÓ ESTUDA	NÃO MORA SÓ	1.00	54.00	2.00	20.00	22.00	22.00	11.00

NUTRICAO	FEMININO	BRANCA	SIM	HETEROSSEXUAL	SEM COMPANHEIRO	SÓ ESTUDA	NÃO MORA SÓ	1.00	58.00	32.00	18.00	12.00	17.00	14.00
NUTRICAO	FEMININO	BRANCA	NÃO	HETEROSSEXUAL	COM COMPANHEIRO	SÓ ESTUDA	NÃO MORA SÓ	1.00	57.00	2.00	12.00	13.00	19.00	18.00
NUTRICAO	FEMININO	BRANCA	NÃO	HETEROSSEXUAL	SEM COMPANHEIRO	SÓ ESTUDA	NÃO MORA SÓ	1.00	44.00	1.00	17.00	9.00	20.00	12.00
NUTRICAO	FEMININO	NÃO BRANCA	SIM	MINORIAS SEXUAIS	SEM COMPANHEIRO	SÓ ESTUDA	NÃO MORA SÓ	1.00	50.00	9.00	19.00	21.00	27.00	7.00
NUTRICAO	FEMININO	NÃO BRANCA	SIM	HETEROSSEXUAL	SEM COMPANHEIRO	SÓ ESTUDA	NÃO MORA SÓ	1.00	52.00	3.00	19.00	20.00	26.00	17.00
NUTRICAO	FEMININO	NÃO BRANCA	SIM	HETEROSSEXUAL	SEM COMPANHEIRO	SÓ ESTUDA	NÃO MORA SÓ	1.00	60.00	1.00	8.00	8.00	11.00	5.00
NUTRICAO	FEMININO	BRANCA	NÃO	HETEROSSEXUAL	SEM COMPANHEIRO	SÓ ESTUDA	NÃO MORA SÓ	1.00	52.00	3.00	20.00	15.00	16.00	7.00
NUTRICAO	FEMININO	NÃO BRANCA	NÃO	HETEROSSEXUAL	COM COMPANHEIRO	SÓ ESTUDA	NÃO MORA SÓ	1.00	55.00	1.00	14.00	9.00	12.00	13.00
NUTRICAO	FEMININO	NÃO BRANCA	SIM	HETEROSSEXUAL	SEM COMPANHEIRO	SÓ ESTUDA	NÃO MORA SÓ	1.00	55.00	9.00	17.00	14.00	15.00	9.00
NUTRICAO	FEMININO	BRANCA	SIM	HETEROSSEXUAL	SEM COMPANHEIRO	SÓ ESTUDA	NÃO MORA SÓ	1.00	69.00	0.00	11.00	9.00	15.00	15.00
NUTRICAO	FEMININO	NÃO BRANCA	NÃO	HETEROSSEXUAL	SEM COMPANHEIRO	SÓ ESTUDA	NÃO MORA SÓ	1.00	30.00	1.00	28.00	15.00	20.00	8.00
NUTRICAO	FEMININO	NÃO BRANCA	NÃO	HETEROSSEXUAL	COM COMPANHEIRO	SÓ ESTUDA	NÃO MORA SÓ	5.00	66.00	0.00	9.00	7.00	9.00	11.00
NUTRICAO	FEMININO	NÃO BRANCA	SIM	HETEROSSEXUAL	SEM COMPANHEIRO	SÓ ESTUDA	NÃO MORA SÓ	1.00	63.00	0.00	11.00	9.00	11.00	7.00
NUTRICAO	FEMININO	NÃO BRANCA	SIM	HETEROSSEXUAL	COM COMPANHEIRO	SÓ ESTUDA	NÃO MORA SÓ	5.00	43.00	0.00	13.00	16.00	17.00	6.00
NUTRICAO	FEMININO	NÃO BRANCA	SIM	HETEROSSEXUAL	COM COMPANHEIRO	SÓ ESTUDA	NÃO MORA SÓ	1.00	48.00	0.00	10.00	9.00	12.00	16.00
NUTRICAO	FEMININO	NÃO BRANCA	SIM	HETEROSSEXUAL	SEM COMPANHEIRO	SÓ ESTUDA		1.00	56.00	0.00	12.00	17.00	23.00	7.00
NUTRICAO	FEMININO	NÃO BRANCA	SIM	HETEROSSEXUAL	SEM COMPANHEIRO	ESTUDA E TRABALHA	NÃO MORA SÓ	1.00	46.00	0.00	17.00	25.00	25.00	15.00
NUTRICAO	FEMININO	NÃO BRANCA	SIM	HETEROSSEXUAL	SEM COMPANHEIRO	SÓ ESTUDA	NÃO MORA SÓ	1.00	39.00	0.00	23.00	25.00	26.00	8.00
NUTRICAO	FEMININO	NÃO BRANCA	NÃO	HETEROSSEXUAL	SEM COMPANHEIRO	SÓ ESTUDA	NÃO MORA SÓ	1.00	55.00	0.00	24.00	19.00	15.00	12.00
PSICOLOGIA	MASCULINO	NÃO BRANCA	NÃO	MINORIAS SEXUAIS	SEM COMPANHEIRO	SÓ ESTUDA	NÃO MORA SÓ	1.00	61.00	0.00	20.00	10.00	15.00	5.00
PSICOLOGIA	FEMININO	NÃO BRANCA	NÃO	MINORIAS SEXUAIS	SEM COMPANHEIRO	SÓ ESTUDA	NÃO MORA SÓ	0.00	40.00	0.00	20.00	10.00	11.00	6.00

PSICOLOGIA	FEMININO	NÃO BRANCA	SIM	HETEROSSEXUAL	COM COMPANHEIRO	SÓ ESTUDA	NÃO MORA SÓ	0.00	29.00	0.00	9.00	16.00	21.00	8.00
PSICOLOGIA	FEMININO	NÃO BRANCA	SIM	HETEROSSEXUAL	SEM COMPANHEIRO	ESTUDA E TRABALHA	NÃO MORA SÓ	0.00	64.00	0.00	9.00	13.00	16.00	14.00
PSICOLOGIA	MASCULINO	NÃO BRANCA	SIM	HETEROSSEXUAL	SEM COMPANHEIRO	SÓ ESTUDA	NÃO MORA SÓ	0.00	58.00	0.00	18.00	16.00	16.00	5.00
PSICOLOGIA	FEMININO	NÃO BRANCA	NÃO	HETEROSSEXUAL	SEM COMPANHEIRO	SÓ ESTUDA	NÃO MORA SÓ	0.00	61.00	0.00	8.00	8.00	9.00	8.00
PSICOLOGIA	FEMININO	NÃO BRANCA	SIM	HETEROSSEXUAL	SEM COMPANHEIRO	SÓ ESTUDA	NÃO MORA SÓ	0.00	40.00	0.00	13.00	11.00	14.00	9.00
PSICOLOGIA	FEMININO	NÃO BRANCA	SIM	HETEROSSEXUAL	SEM COMPANHEIRO	SÓ ESTUDA	MORA SÓ	0.00	40.00	0.00	22.00	14.00	12.00	5.00
PSICOLOGIA	FEMININO	NÃO BRANCA	NÃO	HETEROSSEXUAL	COM COMPANHEIRO	SÓ ESTUDA	NÃO MORA SÓ	0.00	57.00	0.00	11.00	8.00	8.00	11.00
PSICOLOGIA	MASCULINO	NÃO BRANCA	NÃO	HETEROSSEXUAL	SEM COMPANHEIRO	SÓ ESTUDA	NÃO MORA SÓ	0.00	55.00	0.00	10.00	11.00	15.00	5.00
PSICOLOGIA	FEMININO	BRANCA	NÃO	MINORIAS SEXUAIS	COM COMPANHEIRO	SÓ ESTUDA	MORA SÓ	0.00	75.00	0.00	9.00	14.00	15.00	5.00
PSICOLOGIA	FEMININO	BRANCA	NÃO	HETEROSSEXUAL	SEM COMPANHEIRO	SÓ ESTUDA	NÃO MORA SÓ	0.00	38.00	2.00	13.00	14.00	16.00	7.00
PSICOLOGIA	FEMININO	NÃO BRANCA	NÃO	HETEROSSEXUAL	SEM COMPANHEIRO	SÓ ESTUDA	NÃO MORA SÓ	0.00	59.00	1.00	26.00	21.00	20.00	11.00
PSICOLOGIA	FEMININO	BRANCA	NÃO	MINORIAS SEXUAIS	SEM COMPANHEIRO	SÓ ESTUDA	NÃO MORA SÓ	0.00	40.00	7.00	19.00	16.00	24.00	8.00
PSICOLOGIA	FEMININO	NÃO BRANCA	NÃO	HETEROSSEXUAL	SEM COMPANHEIRO	SÓ ESTUDA	NÃO MORA SÓ	0.00	56.00	11.00	19.00	19.00	18.00	8.00
PSICOLOGIA	FEMININO	BRANCA	NÃO	MINORIAS SEXUAIS	SEM COMPANHEIRO	SÓ ESTUDA	NÃO MORA SÓ	0.00	54.00	1.00	17.00	14.00	15.00	6.00
PSICOLOGIA	FEMININO	NÃO BRANCA	SIM	HETEROSSEXUAL	SEM COMPANHEIRO	SÓ ESTUDA	NÃO MORA SÓ	0.00	43.00	13.00	16.00	17.00	22.00	12.00
PSICOLOGIA	MASCULINO	NÃO BRANCA	SIM	MINORIAS SEXUAIS	SEM COMPANHEIRO	ESTUDA E TRABALHA	MORA SÓ	0.00	53.00	0.00	10.00	11.00	15.00	3.00
PSICOLOGIA	FEMININO	BRANCA	SIM	HETEROSSEXUAL	SEM COMPANHEIRO	ESTUDA E TRABALHA	MORA SÓ	0.00	68.00	0.00	7.00	7.00	9.00	6.00
PSICOLOGIA	FEMININO	BRANCA	NÃO	MINORIAS SEXUAIS	SEM COMPANHEIRO	SÓ ESTUDA	NÃO MORA SÓ	0.00	42.00	1.00	25.00	15.00	20.00	8.00
PSICOLOGIA	FEMININO	NÃO BRANCA	SIM	HETEROSSEXUAL	SEM COMPANHEIRO	ESTUDA E TRABALHA	MORA SÓ	0.00	56.00	0.00	10.00	16.00	21.00	12.00
PSICOLOGIA	FEMININO	BRANCA	NÃO	MINORIAS SEXUAIS	COM COMPANHEIRO	ESTUDA E TRABALHA	MORA SÓ	0.00	46.00	19.00	20.00	18.00	21.00	0.00
PSICOLOGIA	FEMININO	BRANCA	SIM	HETEROSSEXUAL	COM COMPANHEIRO	ESTUDA E TRABALHA	NÃO MORA SÓ	0.00	35.00	0.00	12.00	19.00	24.00	1.00

PSICOLOGIA	FEMININO	BRANCA	SIM	HETEROSSEXUAL	SEM COMPANHEIRO	ESTUDA E TRABALHA	NÃO MORA SÓ	0.00	33.00	0.00	17.00	25.00	24.00	15.00
PSICOLOGIA	FEMININO	NÃO BRANCA	NÃO	HETEROSSEXUAL	SEM COMPANHEIRO	SÓ ESTUDA	MORA SÓ	0.00	52.00	4.00	16.00	18.00	20.00	3.00
PSICOLOGIA	FEMININO	NÃO BRANCA	NÃO	HETEROSSEXUAL	SEM COMPANHEIRO	SÓ ESTUDA	NÃO MORA SÓ	0.00	58.00	0.00	13.00	19.00	16.00	6.00
PSICOLOGIA	MASCULINO	NÃO BRANCA	SIM	MINORIAS SEXUAIS	SEM COMPANHEIRO	ESTUDA E TRABALHA	NÃO MORA SÓ	0.00	31.00	7.00	20.00	19.00	21.00	11.00
PSICOLOGIA	FEMININO	BRANCA	NÃO	MINORIAS SEXUAIS	SEM COMPANHEIRO	ESTUDA E TRABALHA	NÃO MORA SÓ	0.00	48.00	19.00	17.00	11.00	14.00	11.00
PSICOLOGIA	FEMININO	NÃO BRANCA	NÃO	MINORIAS SEXUAIS	SEM COMPANHEIRO	SÓ ESTUDA	MORA SÓ	0.00	61.00	0.00	10.00	9.00	12.00	11.00
ENFERMAGEM	MASCULINO	NÃO BRANCA	NÃO	MINORIAS SEXUAIS	SEM COMPANHEIRO	ESTUDA E TRABALHA	NÃO MORA SÓ	0.00	62.00	4.00	10.00	15.00	16.00	4.00
ENFERMAGEM	FEMININO	NÃO BRANCA	SIM	HETEROSSEXUAL	SEM COMPANHEIRO	ESTUDA E TRABALHA	MORA SÓ	0.00	73.00	0.00	8.00	12.00	11.00	8.00
ENFERMAGEM	FEMININO	NÃO BRANCA	NÃO	HETEROSSEXUAL	COM COMPANHEIRO	SÓ ESTUDA	NÃO MORA SÓ	0.00	67.00	0.00	12.00	9.00	12.00	7.00
ENFERMAGEM	MASCULINO	NÃO BRANCA	SIM	MINORIAS SEXUAIS	COM COMPANHEIRO	ESTUDA E TRABALHA	MORA SÓ	0.00	69.00	4.00	14.00	14.00	20.00	9.00
ENFERMAGEM	FEMININO	BRANCA	SIM	HETEROSSEXUAL	COM COMPANHEIRO	SÓ ESTUDA	NÃO MORA SÓ	0.00	63.00	7.00	15.00	15.00	13.00	7.00
ENFERMAGEM	FEMININO	NÃO BRANCA	NÃO	HETEROSSEXUAL	SEM COMPANHEIRO	SÓ ESTUDA	NÃO MORA SÓ	0.00	32.00	0.00	7.00	9.00	12.00	10.00
ENFERMAGEM	FEMININO	BRANCA	NÃO	HETEROSSEXUAL	COM COMPANHEIRO	ESTUDA E TRABALHA	NÃO MORA SÓ	0.00	51.00	11.00	19.00	16.00	18.00	10.00
ENFERMAGEM	FEMININO	BRANCA	NÃO	MINORIAS SEXUAIS	SEM COMPANHEIRO	SÓ ESTUDA	NÃO MORA SÓ	0.00	43.00	0.00	23.00	21.00	19.00	6.00
ENFERMAGEM	MASCULINO	NÃO BRANCA	NÃO	MINORIAS SEXUAIS	SEM COMPANHEIRO	SÓ ESTUDA	NÃO MORA SÓ	0.00	62.00	0.00	18.00	16.00	22.00	10.00
ENFERMAGEM	FEMININO	BRANCA	SIM	HETEROSSEXUAL	SEM COMPANHEIRO	SÓ ESTUDA	NÃO MORA SÓ	0.00	71.00	0.00	10.00	17.00	20.00	6.00
ENFERMAGEM	FEMININO	NÃO BRANCA	SIM	HETEROSSEXUAL	COM COMPANHEIRO	SÓ ESTUDA	NÃO MORA SÓ	0.00	59.00	0.00	14.00	9.00	16.00	12.00
ENFERMAGEM	FEMININO	BRANCA	SIM	HETEROSSEXUAL	SEM COMPANHEIRO	SÓ ESTUDA	NÃO MORA SÓ	0.00	57.00	0.00	8.00	13.00	11.00	13.00
ENFERMAGEM	FEMININO	NÃO BRANCA	NÃO	MINORIAS SEXUAIS	SEM COMPANHEIRO	SÓ ESTUDA	NÃO MORA SÓ	0.00	66.00	0.00	16.00	12.00	14.00	10.00
ENFERMAGEM	FEMININO	NÃO BRANCA	SIM	HETEROSSEXUAL	SEM COMPANHEIRO	SÓ ESTUDA	NÃO MORA SÓ	0.00	47.00	0.00	14.00	10.00	16.00	12.00
ENFERMAGEM	MASCULINO	NÃO BRANCA	SIM	HETEROSSEXUAL	SEM COMPANHEIRO	SÓ ESTUDA	NÃO MORA SÓ	0.00	71.00	0.00	7.00	9.00	10.00	5.00

ENFERMAGEM	MASCULINO	BRANCA	SIM	HETEROSSEXUAL	COM COMPANHEIRO	ESTUDA E TRABALHA	NÃO MORA SÓ	0.00	81.00	0.00	9.00	12.00	13.00	6.00
ENFERMAGEM	MASCULINO	NÃO BRANCA	NÃO	HETEROSSEXUAL	SEM COMPANHEIRO	SÓ ESTUDA		0.00	43.00	0.00	22.00	19.00	25.00	14.00
ENFERMAGEM	FEMININO	BRANCA	SIM	HETEROSSEXUAL	SEM COMPANHEIRO	SÓ ESTUDA	NÃO MORA SÓ	0.00	52.00	2.00	12.00	13.00	12.00	8.00
PSICOLOGIA	FEMININO	NÃO BRANCA	SIM	HETEROSSEXUAL	SEM COMPANHEIRO	ESTUDA E TRABALHA	NÃO MORA SÓ	5.00	67.00	0.00	8.00	8.00	9.00	4.00
PSICOLOGIA	MASCULINO	NÃO BRANCA	NÃO	MINORIAS SEXUAIS	SEM COMPANHEIRO	SÓ ESTUDA	NÃO MORA SÓ	5.00	64.00	0.00	18.00	10.00	17.00	10.00
PSICOLOGIA	MASCULINO	NÃO BRANCA	SIM	HETEROSSEXUAL	COM COMPANHEIRO	ESTUDA E TRABALHA	NÃO MORA SÓ	5.00	56.00	0.00	16.00	13.00	16.00	13.00
PSICOLOGIA	FEMININO	NÃO BRANCA	NÃO	HETEROSSEXUAL	COM COMPANHEIRO	SÓ ESTUDA	NÃO MORA SÓ	5.00	66.00	1.00	16.00	14.00	21.00	10.00
PSICOLOGIA	FEMININO	BRANCA	SIM	HETEROSSEXUAL	SEM COMPANHEIRO	SÓ ESTUDA	NÃO MORA SÓ	5.00	49.00	0.00	10.00	10.00	12.00	8.00
PSICOLOGIA	FEMININO	BRANCA	NÃO	HETEROSSEXUAL	SEM COMPANHEIRO	SÓ ESTUDA	NÃO MORA SÓ	1.00	74.00	0.00	9.00	13.00	12.00	4.00
PSICOLOGIA	FEMININO	NÃO BRANCA	SIM	HETEROSSEXUAL	SEM COMPANHEIRO	SÓ ESTUDA	NÃO MORA SÓ	1.00	51.00	1.00	15.00	10.00	17.00	9.00
PSICOLOGIA	MASCULINO	NÃO BRANCA	SIM	MINORIAS SEXUAIS	SEM COMPANHEIRO	SÓ ESTUDA	MORA SÓ	1.00	55.00	0.00	8.00	7.00	8.00	5.00
PSICOLOGIA	FEMININO	BRANCA	SIM	HETEROSSEXUAL	COM COMPANHEIRO	ESTUDA E TRABALHA	NÃO MORA SÓ	1.00	53.00	0.00	7.00	10.00	10.00	13.00
PSICOLOGIA	FEMININO	BRANCA	SIM	MINORIAS SEXUAIS	COM COMPANHEIRO	SÓ ESTUDA	NÃO MORA SÓ	1.00	59.00	0.00	17.00	19.00	18.00	11.00
PSICOLOGIA	FEMININO	BRANCA	SIM	HETEROSSEXUAL	SEM COMPANHEIRO	ESTUDA E TRABALHA	NÃO MORA SÓ	5.00	28.00	0.00	24.00	20.00	22.00	13.00
PSICOLOGIA	FEMININO	NÃO BRANCA	SIM	HETEROSSEXUAL	SEM COMPANHEIRO	SÓ ESTUDA	NÃO MORA SÓ	5.00	35.00	0.00	9.00	12.00	15.00	14.00
PSICOLOGIA	FEMININO	NÃO BRANCA	SIM	HETEROSSEXUAL	COM COMPANHEIRO	ESTUDA E TRABALHA	NÃO MORA SÓ		62.00	0.00	11.00	12.00	15.00	12.00
PSICOLOGIA	MASCULINO	BRANCA	NÃO	HETEROSSEXUAL	COM COMPANHEIRO	ESTUDA E TRABALHA	NÃO MORA SÓ	5.00	46.00	19.00	22.00	18.00	18.00	13.00
PSICOLOGIA	FEMININO	NÃO BRANCA	NÃO	MINORIAS SEXUAIS	SEM COMPANHEIRO	SÓ ESTUDA	NÃO MORA SÓ	5.00	45.00	23.00	24.00	19.00	20.00	5.00
PSICOLOGIA	FEMININO	NÃO BRANCA	NÃO	HETEROSSEXUAL	COM COMPANHEIRO	ESTUDA E TRABALHA	NÃO MORA SÓ	5.00	48.00	0.00	8.00	13.00	22.00	13.00
PSICOLOGIA	MASCULINO	NÃO BRANCA	SIM	MINORIAS SEXUAIS	COM COMPANHEIRO	SÓ ESTUDA	MORA SÓ	5.00	44.00	19.00	24.00	21.00	24.00	13.00
PSICOLOGIA	FEMININO	BRANCA	SIM	MINORIAS SEXUAIS	COM COMPANHEIRO	SÓ ESTUDA	NÃO MORA SÓ	5.00	32.00	2.00	20.00	9.00	18.00	3.00

PSICOLOGIA	MASCULINO	NÃO BRANCA	NÃO	MINORIAS SEXUAIS	SEM COMPANHEIRO	SÓ ESTUDA	NÃO MORA SÓ	5.00	43.00	2.00	18.00	9.00	18.00	8.00
PSICOLOGIA	FEMININO	NÃO BRANCA	NÃO	HETEROSSEXUAL	SEM COMPANHEIRO	SÓ ESTUDA	MORA SÓ	5.00	66.00	0.00	13.00	8.00	12.00	18.00
PSICOLOGIA	FEMININO	NÃO BRANCA	NÃO	MINORIAS SEXUAIS	SEM COMPANHEIRO	SÓ ESTUDA	MORA SÓ	5.00	48.00	19.00	10.00	17.00	18.00	16.00
PSICOLOGIA	FEMININO	NÃO BRANCA	SIM	HETEROSSEXUAL	SEM COMPANHEIRO	SÓ ESTUDA	NÃO MORA SÓ	5.00	67.00	9.00	17.00	7.00	18.00	6.00
PSICOLOGIA	FEMININO	BRANCA	SIM	HETEROSSEXUAL	COM COMPANHEIRO	ESTUDA E TRABALHA	NÃO MORA SÓ	5.00	49.00	7.00	15.00	17.00	24.00	8.00
PSICOLOGIA	MASCULINO	BRANCA	NÃO	HETEROSSEXUAL	COM COMPANHEIRO	ESTUDA E TRABALHA	MORA SÓ	1.00	22.00	0.00	24.00	26.00	26.00	9.00
PSICOLOGIA	FEMININO	BRANCA	SIM	MINORIAS SEXUAIS	SEM COMPANHEIRO	SÓ ESTUDA	MORA SÓ	1.00	61.00	6.00	20.00	11.00	14.00	11.00
PSICOLOGIA	FEMININO	BRANCA	NÃO	MINORIAS SEXUAIS	SEM COMPANHEIRO	ESTUDA E TRABALHA	NÃO MORA SÓ	1.00	44.00	19.00	23.00	10.00	15.00	9.00
PSICOLOGIA	FEMININO	NÃO BRANCA	SIM	MINORIAS SEXUAIS	COM COMPANHEIRO	SÓ ESTUDA	MORA SÓ	1.00	49.00	10.00	19.00	19.00	16.00	12.00
PSICOLOGIA	FEMININO	NÃO BRANCA	NÃO	MINORIAS SEXUAIS	SEM COMPANHEIRO	SÓ ESTUDA	NÃO MORA SÓ	1.00	72.00	4.00	10.00	10.00	8.00	9.00
PSICOLOGIA	FEMININO	BRANCA	NÃO	MINORIAS SEXUAIS	COM COMPANHEIRO	SÓ ESTUDA	NÃO MORA SÓ	1.00	56.00	0.00	17.00	12.00	19.00	9.00
PSICOLOGIA	FEMININO	NÃO BRANCA	NÃO	MINORIAS SEXUAIS	COM COMPANHEIRO	SÓ ESTUDA	MORA SÓ	1.00	49.00	4.00	15.00	13.00	21.00	13.00
PSICOLOGIA	FEMININO	NÃO BRANCA	SIM	MINORIAS SEXUAIS	SEM COMPANHEIRO	SÓ ESTUDA	NÃO MORA SÓ	1.00	55.00	33.00	7.00	8.00	7.00	5.00
PSICOLOGIA	MASCULINO	NÃO BRANCA	SIM	MINORIAS SEXUAIS	COM COMPANHEIRO	ESTUDA E TRABALHA	NÃO MORA SÓ	1.00	63.00	5.00	12.00	15.00	16.00	15.00
PSICOLOGIA	FEMININO	NÃO BRANCA	SIM	HETEROSSEXUAL	COM COMPANHEIRO	SÓ ESTUDA	NÃO MORA SÓ	1.00	33.00	4.00	20.00	12.00	17.00	17.00
PSICOLOGIA	FEMININO	NÃO BRANCA	SIM	HETEROSSEXUAL	SEM COMPANHEIRO	ESTUDA E TRABALHA	NÃO MORA SÓ	1.00	46.00	4.00	12.00	11.00	14.00	14.00
PSICOLOGIA	FEMININO	BRANCA	NÃO	MINORIAS SEXUAIS	SEM COMPANHEIRO	ESTUDA E TRABALHA	NÃO MORA SÓ	1.00	25.00	23.00	28.00	15.00	24.00	17.00
PSICOLOGIA	FEMININO	BRANCA	SIM	MINORIAS SEXUAIS	COM COMPANHEIRO	SÓ ESTUDA	NÃO MORA SÓ	1.00	54.00	4.00	8.00	7.00	11.00	8.00
PSICOLOGIA	FEMININO	BRANCA	NÃO	MINORIAS SEXUAIS	COM COMPANHEIRO	SÓ ESTUDA	NÃO MORA SÓ	1.00	53.00	13.00	14.00	14.00	15.00	6.00
PSICOLOGIA	FEMININO	NÃO BRANCA	NÃO	HETEROSSEXUAL	COM COMPANHEIRO	ESTUDA E TRABALHA	NÃO MORA SÓ	1.00	64.00	8.00	13.00	12.00	18.00	8.00
PSICOLOGIA	FEMININO	BRANCA	NÃO	MINORIAS SEXUAIS	SEM COMPANHEIRO	SÓ ESTUDA	NÃO MORA SÓ	1.00	50.00	9.00	19.00	21.00	19.00	7.00

PSICOLOGIA	FEMININO	NÃO BRANCA	NÃO	HETEROSSEXUAL	SEM COMPANHEIRO	SÓ ESTUDA	NÃO MORA SÓ	1.00	42.00	0.00	16.00	14.00	26.00	9.00
PSICOLOGIA	FEMININO	BRANCA	SIM	MINORIAS SEXUAIS	SEM COMPANHEIRO	SÓ ESTUDA	NÃO MORA SÓ	1.00	45.00	22.00	21.00	21.00	21.00	2.00
PSICOLOGIA		BRANCA	NÃO	MINORIAS SEXUAIS	SEM COMPANHEIRO	SÓ ESTUDA	NÃO MORA SÓ	1.00	50.00	0.00	11.00	7.00	8.00	9.00
PSICOLOGIA	FEMININO	BRANCA	NÃO	HETEROSSEXUAL		SÓ ESTUDA	NÃO MORA SÓ	1.00	49.00	2.00	25.00	10.00	12.00	9.00
PSICOLOGIA	MASCULINO	NÃO BRANCA	NÃO	HETEROSSEXUAL	COM COMPANHEIRO	ESTUDA E TRABALHA	NÃO MORA SÓ	1.00	59.00	0.00	7.00	11.00	12.00	11.00
PSICOLOGIA	MASCULINO	BRANCA	NÃO	MINORIAS SEXUAIS	SEM COMPANHEIRO	SÓ ESTUDA	MORA SÓ	1.00	29.00	23.00	28.00	19.00	27.00	7.00
PSICOLOGIA	FEMININO	NÃO BRANCA	NÃO	MINORIAS SEXUAIS	SEM COMPANHEIRO	ESTUDA E TRABALHA	NÃO MORA SÓ		62.00	0.00	18.00	11.00	15.00	11.00
PSICOLOGIA	FEMININO	BRANCA	NÃO	HETEROSSEXUAL	SEM COMPANHEIRO	ESTUDA E TRABALHA	NÃO MORA SÓ	3.00	30.00	0.00	11.00	7.00	19.00	5.00
PSICOLOGIA	FEMININO	NÃO BRANCA	SIM	HETEROSSEXUAL	SEM COMPANHEIRO	SÓ ESTUDA	NÃO MORA SÓ	3.00	52.00	0.00	13.00	10.00	20.00	12.00
PSICOLOGIA	FEMININO	BRANCA	NÃO	HETEROSSEXUAL	COM COMPANHEIRO	SÓ ESTUDA	NÃO MORA SÓ	3.00	53.00	0.00	11.00	7.00	11.00	7.00
PSICOLOGIA	FEMININO	NÃO BRANCA	NÃO	HETEROSSEXUAL	SEM COMPANHEIRO	SÓ ESTUDA	NÃO MORA SÓ	3.00	50.00	1.00	10.00	11.00	12.00	8.00
PSICOLOGIA	FEMININO	BRANCA	NÃO	HETEROSSEXUAL	COM COMPANHEIRO	ESTUDA E TRABALHA	NÃO MORA SÓ	3.00	70.00	0.00	7.00	8.00	9.00	6.00
PSICOLOGIA	FEMININO	BRANCA	NÃO	HETEROSSEXUAL	SEM COMPANHEIRO	SÓ ESTUDA	NÃO MORA SÓ	3.00	61.00	1.00	7.00	7.00	8.00	3.00
PSICOLOGIA	FEMININO	NÃO BRANCA	SIM	HETEROSSEXUAL	SEM COMPANHEIRO	SÓ ESTUDA	MORA SÓ	3.00	51.00	5.00	21.00	19.00	18.00	12.00
PSICOLOGIA	FEMININO	NÃO BRANCA	NÃO	MINORIAS SEXUAIS	COM COMPANHEIRO	ESTUDA E TRABALHA	NÃO MORA SÓ		50.00	6.00	12.00	6.00	27.00	13.00
PSICOLOGIA	FEMININO	BRANCA	NÃO	HETEROSSEXUAL	SEM COMPANHEIRO	SÓ ESTUDA	NÃO MORA SÓ	3.00	70.00	0.00	9.00	12.00	11.00	12.00
PSICOLOGIA	FEMININO	NÃO BRANCA	NÃO	HETEROSSEXUAL	COM COMPANHEIRO	ESTUDA E TRABALHA	MORA SÓ	3.00	57.00	0.00	8.00	9.00	13.00	13.00
PSICOLOGIA	FEMININO	NÃO BRANCA	NÃO	MINORIAS SEXUAIS	COM COMPANHEIRO	SÓ ESTUDA	NÃO MORA SÓ	3.00	70.00	5.00	9.00	7.00	12.00	9.00
PSICOLOGIA	FEMININO	NÃO BRANCA	NÃO		SEM COMPANHEIRO	SÓ ESTUDA	NÃO MORA SÓ	3.00	58.00	0.00	8.00	11.00	11.00	11.00
PSICOLOGIA	MASCULINO	NÃO BRANCA	NÃO	HETEROSSEXUAL	SEM COMPANHEIRO	SÓ ESTUDA	NÃO MORA SÓ	3.00	69.00	0.00	7.00	8.00	8.00	7.00
PSICOLOGIA	FEMININO	NÃO BRANCA	SIM	HETEROSSEXUAL	COM COMPANHEIRO	ESTUDA E TRABALHA	NÃO MORA SÓ	3.00	20.00	0.00	10.00	12.00	24.00	0.00

PSICOLOGIA	FEMININO	NÃO BRANCA	NÃO	HETEROSSEXUAL	SEM COMPANHEIRO	SÓ ESTUDA	NÃO MORA SÓ	3.00	42.00	1.00	25.00	12.00	17.00	14.00
PSICOLOGIA	FEMININO	NÃO BRANCA	SIM	HETEROSSEXUAL	COM COMPANHEIRO	SÓ ESTUDA	NÃO MORA SÓ	3.00	62.00	4.00	16.00	12.00	16.00	11.00
PSICOLOGIA	FEMININO	BRANCA	NÃO	HETEROSSEXUAL	COM COMPANHEIRO	SÓ ESTUDA	NÃO MORA SÓ	3.00	42.00	4.00	9.00	13.00	12.00	8.00
PSICOLOGIA	FEMININO	NÃO BRANCA	SIM	HETEROSSEXUAL	COM COMPANHEIRO	SÓ ESTUDA	NÃO MORA SÓ	3.00	50.00	0.00	24.00	16.00	21.00	16.00
PSICOLOGIA	MASCULINO	BRANCA	NÃO	MINORIAS SEXUAIS	COM COMPANHEIRO	ESTUDA E TRABALHA	MORA SÓ	3.00	30.00	7.00	17.00	16.00	22.00	17.00
PSICOLOGIA	MASCULINO	NÃO BRANCA	NÃO	HETEROSSEXUAL	SEM COMPANHEIRO	ESTUDA E TRABALHA	NÃO MORA SÓ	3.00	46.00	19.00	28.00	22.00	22.00	7.00
PSICOLOGIA	MASCULINO	BRANCA	SIM	MINORIAS SEXUAIS	COM COMPANHEIRO	ESTUDA E TRABALHA	NÃO MORA SÓ	3.00	25.00	0.00	18.00	20.00	20.00	6.00
PSICOLOGIA	FEMININO	NÃO BRANCA	SIM	HETEROSSEXUAL	SEM COMPANHEIRO	ESTUDA E TRABALHA	NÃO MORA SÓ	3.00	61.00	0.00	14.00	24.00	17.00	8.00
PSICOLOGIA	FEMININO	BRANCA	SIM	MINORIAS SEXUAIS	SEM COMPANHEIRO	ESTUDA E TRABALHA	MORA SÓ	3.00	60.00	14.00	9.00	12.00	12.00	8.00
PSICOLOGIA	FEMININO	NÃO BRANCA	SIM		SEM COMPANHEIRO	SÓ ESTUDA	NÃO MORA SÓ	3.00	67.00	19.00	17.00	7.00	10.00	9.00
PSICOLOGIA	FEMININO	BRANCA	NÃO	MINORIAS SEXUAIS	SEM COMPANHEIRO	SÓ ESTUDA	NÃO MORA SÓ	3.00	69.00	0.00	8.00	7.00	10.00	17.00
PSICOLOGIA	FEMININO	NÃO BRANCA	NÃO	HETEROSSEXUAL	COM COMPANHEIRO		NÃO MORA SÓ	3.00	56.00	0.00	10.00	16.00	17.00	13.00
PSICOLOGIA	FEMININO	BRANCA	SIM	HETEROSSEXUAL	COM COMPANHEIRO	SÓ ESTUDA	NÃO MORA SÓ	3.00	68.00	0.00	7.00	7.00	8.00	6.00
PSICOLOGIA	FEMININO	NÃO BRANCA	SIM	MINORIAS SEXUAIS	SEM COMPANHEIRO	ESTUDA E TRABALHA	MORA SÓ	3.00	25.00	3.00	19.00	22.00	25.00	15.00
PSICOLOGIA	FEMININO	BRANCA	NÃO	MINORIAS SEXUAIS	COM COMPANHEIRO	SÓ ESTUDA	NÃO MORA SÓ	3.00	52.00	1.00	12.00	11.00	15.00	7.00
PSICOLOGIA	FEMININO	NÃO BRANCA	NÃO	HETEROSSEXUAL	SEM COMPANHEIRO	ESTUDA E TRABALHA	NÃO MORA SÓ	3.00	35.00	0.00	12.00	12.00	14.00	12.00
PSICOLOGIA	FEMININO	BRANCA	SIM	HETEROSSEXUAL	COM COMPANHEIRO	SÓ ESTUDA	NÃO MORA SÓ	3.00	46.00	0.00	11.00	15.00	14.00	14.00
PSICOLOGIA	FEMININO	BRANCA	SIM	MINORIAS SEXUAIS	COM COMPANHEIRO	SÓ ESTUDA	NÃO MORA SÓ	1.00	64.00	0.00	17.00	18.00	18.00	16.00
PSICOLOGIA	FEMININO	BRANCA	NÃO	HETEROSSEXUAL	SEM COMPANHEIRO	SÓ ESTUDA	NÃO MORA SÓ	1.00	52.00	3.00	18.00	12.00	27.00	12.00
PSICOLOGIA	FEMININO	BRANCA	NÃO	MINORIAS SEXUAIS	COM COMPANHEIRO	ESTUDA E TRABALHA	NÃO MORA SÓ	1.00	35.00	0.00	14.00	8.00	16.00	8.00
PSICOLOGIA	MASCULINO	NÃO BRANCA	NÃO	HETEROSSEXUAL	SEM COMPANHEIRO	ESTUDA E TRABALHA	NÃO MORA SÓ	1.00	44.00	2.00	19.00	19.00	25.00	7.00

PSICOLOGIA	FEMININO	NÃO BRANCA	NÃO	MINORIAS SEXUAIS	SEM COMPANHEIRO	ESTUDA E TRABALHA	NÃO MORA SÓ	1.00	43.00	0.00	12.00	14.00	15.00	14.00
PSICOLOGIA	FEMININO	NÃO BRANCA	SIM	HETEROSSEXUAL	SEM COMPANHEIRO	SÓ ESTUDA	NÃO MORA SÓ	1.00	61.00	0.00	13.00	14.00	16.00	7.00
PSICOLOGIA	FEMININO	BRANCA	SIM	HETEROSSEXUAL	SEM COMPANHEIRO	SÓ ESTUDA	NÃO MORA SÓ	1.00	41.00	0.00	13.00	10.00	18.00	12.00
PSICOLOGIA	FEMININO	BRANCA	NÃO	MINORIAS SEXUAIS	SEM COMPANHEIRO	ESTUDA E TRABALHA	NÃO MORA SÓ	1.00	30.00	17.00	21.00	15.00	25.00	7.00
PSICOLOGIA	MASCULINO	BRANCA	NÃO	MINORIAS SEXUAIS	SEM COMPANHEIRO	ESTUDA E TRABALHA	NÃO MORA SÓ	1.00	28.00	7.00	17.00	24.00	24.00	23.00
PSICOLOGIA	FEMININO	NÃO BRANCA	SIM	HETEROSSEXUAL	SEM COMPANHEIRO	SÓ ESTUDA	NÃO MORA SÓ	1.00	43.00	19.00	18.00	18.00	21.00	10.00
PSICOLOGIA	FEMININO	NÃO BRANCA	NÃO	MINORIAS SEXUAIS	SEM COMPANHEIRO	SÓ ESTUDA	NÃO MORA SÓ	1.00	41.00	19.00	19.00	27.00	22.00	12.00
PSICOLOGIA	FEMININO	NÃO BRANCA	SIM	HETEROSSEXUAL	COM COMPANHEIRO	SÓ ESTUDA	NÃO MORA SÓ	1.00	31.00	4.00	20.00	18.00	22.00	14.00
PSICOLOGIA	FEMININO	NÃO BRANCA	NÃO	MINORIAS SEXUAIS	SEM COMPANHEIRO	ESTUDA E TRABALHA	NÃO MORA SÓ	1.00	49.00	9.00	11.00	18.00	20.00	8.00
PSICOLOGIA	MASCULINO	BRANCA	NÃO	HETEROSSEXUAL	SEM COMPANHEIRO	ESTUDA E TRABALHA	NÃO MORA SÓ	1.00	63.00	9.00	20.00	8.00	11.00	6.00
PSICOLOGIA	MASCULINO	NÃO BRANCA	NÃO	MINORIAS SEXUAIS	SEM COMPANHEIRO	SÓ ESTUDA	NÃO MORA SÓ	1.00	43.00	11.00	15.00	11.00	20.00	10.00
PSICOLOGIA	MASCULINO	BRANCA	NÃO	MINORIAS SEXUAIS	SEM COMPANHEIRO	ESTUDA E TRABALHA	NÃO MORA SÓ	1.00	57.00	2.00	10.00	12.00	15.00	14.00
PSICOLOGIA	MASCULINO	BRANCA	NÃO	HETEROSSEXUAL	SEM COMPANHEIRO	SÓ ESTUDA	NÃO MORA SÓ	1.00	62.00	17.00	19.00	10.00	9.00	8.00
PSICOLOGIA	FEMININO	BRANCA	NÃO	HETEROSSEXUAL	COM COMPANHEIRO	ESTUDA E TRABALHA	NÃO MORA SÓ	1.00	47.00	23.00	21.00	13.00	22.00	7.00
PSICOLOGIA	FEMININO	NÃO BRANCA		HETEROSSEXUAL	COM COMPANHEIRO	SÓ ESTUDA	NÃO MORA SÓ	1.00	49.00	0.00	9.00	12.00	17.00	12.00
ENFERMAGEM	FEMININO	BRANCA	SIM	HETEROSSEXUAL	SEM COMPANHEIRO	SÓ ESTUDA	NÃO MORA SÓ	0.00	26.00	0.00	8.00	9.00	19.00	
ENFERMAGEM	MASCULINO	NÃO BRANCA	NÃO	MINORIAS SEXUAIS	SEM COMPANHEIRO	SÓ ESTUDA	MORA SÓ	0.00	50.00	19.00	21.00	23.00	20.00	7.00
ENFERMAGEM	FEMININO	NÃO BRANCA	SIM	HETEROSSEXUAL	SEM COMPANHEIRO	SÓ ESTUDA	NÃO MORA SÓ	0.00	52.00	6.00	12.00	13.00	16.00	12.00
ENFERMAGEM	FEMININO	NÃO BRANCA	SIM	MINORIAS SEXUAIS	SEM COMPANHEIRO	SÓ ESTUDA	NÃO MORA SÓ	0.00	35.00	2.00	25.00	16.00	18.00	12.00
ENFERMAGEM	FEMININO	NÃO BRANCA	NÃO	HETEROSSEXUAL	COM COMPANHEIRO	SÓ ESTUDA	NÃO MORA SÓ	0.00	57.00	4.00	20.00	24.00	22.00	6.00
ENFERMAGEM	MASCULINO	BRANCA	NÃO	MINORIAS SEXUAIS	SEM COMPANHEIRO	SÓ ESTUDA	NÃO MORA SÓ	0.00	46.00	19.00	19.00	14.00	13.00	9.00

ENFERMAGEM	FEMININO	NÃO BRANCA	NÃO	HETEROSSEXUAL	COM COMPANHEIRO	SÓ ESTUDA	NÃO MORA SÓ	1.00	52.00	19.00	11.00	13.00	8.00	12.00
ENFERMAGEM	FEMININO	NÃO BRANCA	SIM	HETEROSSEXUAL	SEM COMPANHEIRO	ESTUDA E TRABALHA	NÃO MORA SÓ	0.00	50.00	0.00	16.00	17.00	20.00	8.00
ENFERMAGEM	FEMININO	NÃO BRANCA	SIM	HETEROSSEXUAL	COM COMPANHEIRO	SÓ ESTUDA	NÃO MORA SÓ	0.00	76.00	0.00	8.00	10.00	11.00	11.00
ENFERMAGEM	FEMININO	NÃO BRANCA	SIM	HETEROSSEXUAL	SEM COMPANHEIRO	SÓ ESTUDA	NÃO MORA SÓ	0.00	66.00	0.00	9.00	11.00	10.00	4.00
ENFERMAGEM	MASCULINO	BRANCA	NÃO	HETEROSSEXUAL	SEM COMPANHEIRO	SÓ ESTUDA	NÃO MORA SÓ	1.00	62.00	0.00	10.00	14.00	14.00	2.00
ENFERMAGEM	MASCULINO	NÃO BRANCA	SIM	MINORIAS SEXUAIS	SEM COMPANHEIRO	ESTUDA E TRABALHA	NÃO MORA SÓ	1.00	58.00	0.00	12.00	10.00	13.00	3.00
ENFERMAGEM	MASCULINO	BRANCA	SIM	MINORIAS SEXUAIS	SEM COMPANHEIRO	SÓ ESTUDA	NÃO MORA SÓ	0.00	56.00	0.00	11.00	14.00	21.00	14.00
ENFERMAGEM	FEMININO	BRANCA	SIM	HETEROSSEXUAL	SEM COMPANHEIRO	SÓ ESTUDA	NÃO MORA SÓ	0.00	44.00	0.00	13.00	19.00	19.00	12.00
ENFERMAGEM	FEMININO	NÃO BRANCA	SIM	HETEROSSEXUAL	SEM COMPANHEIRO	SÓ ESTUDA	NÃO MORA SÓ	0.00	47.00	1.00	24.00	20.00	21.00	14.00
ENFERMAGEM	MASCULINO	NÃO BRANCA	NÃO	MINORIAS SEXUAIS	COM COMPANHEIRO	ESTUDA E TRABALHA	NÃO MORA SÓ	0.00	78.00	0.00	9.00	14.00	19.00	3.00
ENFERMAGEM	FEMININO	NÃO BRANCA	SIM	HETEROSSEXUAL	SEM COMPANHEIRO	SÓ ESTUDA	NÃO MORA SÓ	0.00	63.00	0.00	10.00	10.00	15.00	8.00
ENFERMAGEM	FEMININO	NÃO BRANCA	SIM	HETEROSSEXUAL	SEM COMPANHEIRO	SÓ ESTUDA	NÃO MORA SÓ	0.00	56.00	0.00	11.00	7.00	13.00	5.00
ENFERMAGEM	FEMININO	NÃO BRANCA	SIM	HETEROSSEXUAL	COM COMPANHEIRO	ESTUDA E TRABALHA	NÃO MORA SÓ	1.00	46.00	4.00	12.00	20.00	23.00	7.00
ENFERMAGEM	FEMININO	BRANCA	NÃO	HETEROSSEXUAL	COM COMPANHEIRO	SÓ ESTUDA	NÃO MORA SÓ	1.00	33.00	23.00	24.00	26.00	24.00	18.00
ENFERMAGEM	FEMININO	BRANCA	SIM	HETEROSSEXUAL	SEM COMPANHEIRO	SÓ ESTUDA	NÃO MORA SÓ	1.00	57.00	8.00	21.00	14.00	21.00	10.00
ENFERMAGEM	FEMININO	BRANCA	SIM	HETEROSSEXUAL	COM COMPANHEIRO	SÓ ESTUDA	NÃO MORA SÓ	1.00	29.00	2.00	20.00	18.00	26.00	18.00
ENFERMAGEM	FEMININO	BRANCA	NÃO	MINORIAS SEXUAIS	SEM COMPANHEIRO	SÓ ESTUDA	NÃO MORA SÓ	1.00	46.00	23.00	14.00	17.00	26.00	8.00
ENFERMAGEM	FEMININO	NÃO BRANCA	SIM	HETEROSSEXUAL	SEM COMPANHEIRO	ESTUDA E TRABALHA	NÃO MORA SÓ	1.00	46.00	0.00	10.00	9.00	17.00	17.00
ENFERMAGEM	FEMININO	NÃO BRANCA	SIM	HETEROSSEXUAL	SEM COMPANHEIRO	SÓ ESTUDA	NÃO MORA SÓ	1.00	43.00	0.00	21.00	10.00	21.00	13.00
ENFERMAGEM	FEMININO	NÃO BRANCA	SIM	HETEROSSEXUAL	COM COMPANHEIRO	ESTUDA E TRABALHA	NÃO MORA SÓ	1.00	50.00	0.00	15.00	17.00	20.00	12.00
ENFERMAGEM	MASCULINO	NÃO BRANCA	NÃO	HETEROSSEXUAL	SEM COMPANHEIRO	ESTUDA E TRABALHA	NÃO MORA SÓ	1.00	60.00	0.00	13.00	13.00	13.00	11.00

ENFERMAGEM	MASCULINO	BRANCA	NÃO	MINORIAS SEXUAIS	SEM COMPANHEIRO	SÓ ESTUDA	NÃO MORA SÓ	1.00	54.00	0.00	13.00	12.00	16.00	11.00
ENFERMAGEM	FEMININO	BRANCA	SIM	HETEROSSEXUAL	SEM COMPANHEIRO	SÓ ESTUDA	NÃO MORA SÓ	1.00	68.00	0.00	14.00	10.00	13.00	13.00
ENFERMAGEM	FEMININO	NÃO BRANCA	SIM	HETEROSSEXUAL	SEM COMPANHEIRO	SÓ ESTUDA	NÃO MORA SÓ	0.00	52.00	0.00	12.00	8.00	12.00	6.00
ENFERMAGEM	MASCULINO	BRANCA	SIM	HETEROSSEXUAL	COM COMPANHEIRO	SÓ ESTUDA	NÃO MORA SÓ	1.00	63.00	0.00	14.00	16.00	19.00	9.00
ENFERMAGEM	MASCULINO	NÃO BRANCA	NÃO	HETEROSSEXUAL	SEM COMPANHEIRO	SÓ ESTUDA	NÃO MORA SÓ	1.00	46.00	0.00	16.00	18.00	18.00	12.00
ENFERMAGEM	FEMININO	BRANCA	SIM	HETEROSSEXUAL	SEM COMPANHEIRO	SÓ ESTUDA	NÃO MORA SÓ	5.00	64.00	9.00	10.00	9.00	9.00	7.00
ENFERMAGEM	FEMININO	BRANCA	SIM	HETEROSSEXUAL	SEM COMPANHEIRO	SÓ ESTUDA	NÃO MORA SÓ	5.00	45.00	0.00	14.00	15.00	21.00	10.00
ENFERMAGEM	FEMININO	BRANCA	NÃO	HETEROSSEXUAL	COM COMPANHEIRO	SÓ ESTUDA	MORA SÓ	5.00	52.00	0.00	17.00	13.00	20.00	11.00
ENFERMAGEM	FEMININO	BRANCA	SIM	MINORIAS SEXUAIS	SEM COMPANHEIRO	ESTUDA E TRABALHA	MORA SÓ	5.00	16.00	33.00	23.00	21.00	24.00	16.00
ENFERMAGEM	FEMININO	BRANCA	NÃO	HETEROSSEXUAL	COM COMPANHEIRO	SÓ ESTUDA	NÃO MORA SÓ	1.00	42.00	11.00	18.00	24.00	28.00	13.00
ENFERMAGEM	FEMININO	BRANCA	SIM	HETEROSSEXUAL	COM COMPANHEIRO	SÓ ESTUDA	NÃO MORA SÓ	1.00	43.00	9.00	24.00	23.00	23.00	16.00
ENFERMAGEM	FEMININO	BRANCA	SIM	HETEROSSEXUAL	COM COMPANHEIRO	SÓ ESTUDA	NÃO MORA SÓ	1.00	49.00	9.00	13.00	13.00	16.00	6.00
ENFERMAGEM	FEMININO	NÃO BRANCA	NÃO	MINORIAS SEXUAIS	COM COMPANHEIRO	SÓ ESTUDA	NÃO MORA SÓ	5.00	43.00	21.00	28.00	23.00	19.00	7.00
ENFERMAGEM	FEMININO	NÃO BRANCA	SIM	HETEROSSEXUAL	SEM COMPANHEIRO	ESTUDA E TRABALHA	NÃO MORA SÓ	1.00	17.00	4.00	20.00	20.00	20.00	0.00
ENFERMAGEM	FEMININO	NÃO BRANCA	SIM	HETEROSSEXUAL	SEM COMPANHEIRO	SÓ ESTUDA	NÃO MORA SÓ	1.00	58.00	0.00	14.00	12.00	12.00	8.00
ENFERMAGEM	FEMININO	NÃO BRANCA	SIM	HETEROSSEXUAL	SEM COMPANHEIRO	SÓ ESTUDA	NÃO MORA SÓ	5.00	81.00	0.00	15.00	14.00	18.00	8.00
ENFERMAGEM	FEMININO	NÃO BRANCA	NÃO	MINORIAS SEXUAIS	COM COMPANHEIRO	SÓ ESTUDA	NÃO MORA SÓ	1.00	56.00	0.00	20.00	12.00	16.00	10.00
ENFERMAGEM	FEMININO	NÃO BRANCA	SIM	HETEROSSEXUAL	COM COMPANHEIRO	SÓ ESTUDA	NÃO MORA SÓ	1.00	67.00	0.00	10.00	12.00	13.00	6.00
ENFERMAGEM	FEMININO	BRANCA	SIM	HETEROSSEXUAL	SEM COMPANHEIRO	SÓ ESTUDA	NÃO MORA SÓ	1.00	66.00	0.00	9.00	10.00	16.00	15.00
ENFERMAGEM	FEMININO	BRANCA	SIM	HETEROSSEXUAL	COM COMPANHEIRO	SÓ ESTUDA	NÃO MORA SÓ	1.00	59.00	0.00	8.00	13.00	14.00	16.00
ENFERMAGEM	FEMININO	NÃO BRANCA	SIM	HETEROSSEXUAL	COM COMPANHEIRO	SÓ ESTUDA	NÃO MORA SÓ	1.00	56.00	0.00	12.00	18.00	19.00	12.00

ENFERMAGEM	FEMININO	NÃO BRANCA	SIM	HETEROSSEXUAL	SEM COMPANHEIRO	SÓ ESTUDA	NÃO MORA SÓ	1.00	62.00	0.00	13.00	9.00	13.00	10.00
ENFERMAGEM	FEMININO	NÃO BRANCA	SIM	HETEROSSEXUAL	SEM COMPANHEIRO	SÓ ESTUDA	NÃO MORA SÓ	1.00	38.00	3.00	28.00	23.00	27.00	10.00
ENFERMAGEM	MASCULINO	BRANCA	SIM	MINORIAS SEXUAIS	COM COMPANHEIRO	SÓ ESTUDA	NÃO MORA SÓ	1.00	49.00	0.00	11.00	10.00	19.00	7.00
ENFERMAGEM	FEMININO	NÃO BRANCA	SIM	MINORIAS SEXUAIS	SEM COMPANHEIRO	SÓ ESTUDA	NÃO MORA SÓ	1.00	56.00	4.00	14.00	28.00	22.00	10.00
ENFERMAGEM	FEMININO	BRANCA	NÃO	HETEROSSEXUAL	SEM COMPANHEIRO	SÓ ESTUDA	NÃO MORA SÓ	1.00	62.00	5.00	13.00	12.00	19.00	13.00
ENFERMAGEM	MASCULINO	BRANCA	NÃO	HETEROSSEXUAL	SEM COMPANHEIRO	ESTUDA E TRABALHA	NÃO MORA SÓ	1.00	48.00	7.00	16.00	12.00	9.00	10.00
ENFERMAGEM	FEMININO	BRANCA	SIM	HETEROSSEXUAL	SEM COMPANHEIRO	ESTUDA E TRABALHA	NÃO MORA SÓ	1.00	59.00	0.00	21.00	16.00	15.00	6.00
ENFERMAGEM	FEMININO	BRANCA	SIM	HETEROSSEXUAL	SEM COMPANHEIRO	ESTUDA E TRABALHA	NÃO MORA SÓ	1.00	50.00	6.00	17.00	13.00	18.00	12.00
ENFERMAGEM	FEMININO	BRANCA	SIM	MINORIAS SEXUAIS	SEM COMPANHEIRO	SÓ ESTUDA	NÃO MORA SÓ	1.00	58.00	13.00	9.00	8.00	10.00	18.00
ENFERMAGEM	MASCULINO	NÃO BRANCA	SIM	MINORIAS SEXUAIS	SEM COMPANHEIRO	SÓ ESTUDA	MORA SÓ	1.00	35.00	17.00	14.00	24.00	24.00	17.00
ENFERMAGEM	FEMININO	NÃO BRANCA	SIM	HETEROSSEXUAL	SEM COMPANHEIRO	SÓ ESTUDA	NÃO MORA SÓ	1.00	61.00	4.00	7.00	8.00	17.00	11.00
ENFERMAGEM	FEMININO	BRANCA	NÃO	HETEROSSEXUAL	SEM COMPANHEIRO	SÓ ESTUDA	NÃO MORA SÓ	1.00	66.00	1.00	15.00	15.00	21.00	11.00
ENFERMAGEM	FEMININO	BRANCA	SIM	HETEROSSEXUAL	COM COMPANHEIRO	SÓ ESTUDA	NÃO MORA SÓ	1.00	53.00	0.00	15.00	12.00	17.00	10.00
ENFERMAGEM	FEMININO	BRANCA	SIM	HETEROSSEXUAL	SEM COMPANHEIRO	SÓ ESTUDA	NÃO MORA SÓ	1.00	62.00	1.00	14.00	8.00	17.00	12.00
ENFERMAGEM	FEMININO	NÃO BRANCA	SIM	HETEROSSEXUAL	COM COMPANHEIRO	SÓ ESTUDA	NÃO MORA SÓ	1.00	56.00	1.00	14.00	12.00	20.00	10.00
ENFERMAGEM	FEMININO	BRANCA	SIM	HETEROSSEXUAL	COM COMPANHEIRO	SÓ ESTUDA	NÃO MORA SÓ	1.00	71.00	0.00	7.00	7.00	11.00	12.00
ENFERMAGEM	FEMININO	NÃO BRANCA	SIM	HETEROSSEXUAL	SEM COMPANHEIRO	SÓ ESTUDA	NÃO MORA SÓ	1.00	63.00	0.00	12.00	10.00	13.00	3.00
ENFERMAGEM	FEMININO	BRANCA	SIM	HETEROSSEXUAL	SEM COMPANHEIRO	SÓ ESTUDA	NÃO MORA SÓ	1.00	71.00	0.00	11.00	7.00	10.00	12.00
ENFERMAGEM	MASCULINO	NÃO BRANCA	SIM	HETEROSSEXUAL	SEM COMPANHEIRO	SÓ ESTUDA	NÃO MORA SÓ	1.00	76.00	0.00	12.00	13.00	17.00	15.00
NUTRICAO	FEMININO	NÃO BRANCA	SIM	HETEROSSEXUAL	SEM COMPANHEIRO	SÓ ESTUDA	MORA SÓ	5.00	46.00	17.00	24.00	16.00	17.00	11.00
NUTRICAO	FEMININO	BRANCA	SIM	HETEROSSEXUAL	SEM COMPANHEIRO		NÃO MORA SÓ	5.00	34.00	0.00	15.00	11.00	12.00	11.00

NUTRICAO	FEMININO	NÃO BRANCA	NÃO	HETEROSSEXUAL	COM COMPANHEIRO	SÓ ESTUDA	NÃO MORA SÓ	5.00	55.00	19.00	19.00	14.00	18.00	8.00
NUTRICAO	FEMININO	BRANCA	SIM	HETEROSSEXUAL	SEM COMPANHEIRO	SÓ ESTUDA	NÃO MORA SÓ	5.00	54.00	0.00	12.00	10.00	12.00	12.00
NUTRICAO	FEMININO	BRANCA	NÃO	HETEROSSEXUAL	COM COMPANHEIRO	SÓ ESTUDA	NÃO MORA SÓ	5.00	43.00	16.00	15.00	15.00	15.00	13.00
NUTRICAO	FEMININO	BRANCA	SIM	HETEROSSEXUAL	COM COMPANHEIRO	SÓ ESTUDA	NÃO MORA SÓ	5.00	21.00	18.00	28.00	16.00	25.00	12.00
NUTRICAO	FEMININO	BRANCA	NÃO	HETEROSSEXUAL	COM COMPANHEIRO	ESTUDA E TRABALHA	NÃO MORA SÓ	5.00	45.00	0.00	12.00	10.00	25.00	10.00
NUTRICAO	FEMININO	BRANCA	SIM	HETEROSSEXUAL	SEM COMPANHEIRO	SÓ ESTUDA	MORA SÓ	5.00	42.00	1.00	28.00	18.00	21.00	14.00
NUTRICAO	MASCULINO	NÃO BRANCA	NÃO	HETEROSSEXUAL	SEM COMPANHEIRO	SÓ ESTUDA	MORA SÓ	5.00	42.00	23.00	25.00	9.00	18.00	4.00
NUTRICAO	FEMININO	NÃO BRANCA	SIM	HETEROSSEXUAL	SEM COMPANHEIRO	SÓ ESTUDA	NÃO MORA SÓ	5.00	67.00	0.00	9.00	14.00	13.00	6.00
NUTRICAO	FEMININO	NÃO BRANCA	SIM	HETEROSSEXUAL	COM COMPANHEIRO	SÓ ESTUDA	NÃO MORA SÓ	5.00	47.00	0.00	18.00	9.00	19.00	11.00
NUTRICAO	FEMININO	NÃO BRANCA	NÃO	HETEROSSEXUAL	SEM COMPANHEIRO	SÓ ESTUDA	NÃO MORA SÓ	5.00	45.00	1.00	16.00	13.00	14.00	8.00
NUTRICAO	FEMININO	NÃO BRANCA	SIM	HETEROSSEXUAL	SEM COMPANHEIRO	SÓ ESTUDA	NÃO MORA SÓ	5.00	69.00	1.00	10.00	9.00	13.00	11.00
NUTRICAO	FEMININO	NÃO BRANCA	SIM	HETEROSSEXUAL	SEM COMPANHEIRO	SÓ ESTUDA	NÃO MORA SÓ	5.00	74.00	0.00	7.00	9.00	9.00	11.00
NUTRICAO	FEMININO	NÃO BRANCA	NÃO	HETEROSSEXUAL	SEM COMPANHEIRO	SÓ ESTUDA	NÃO MORA SÓ	5.00	66.00	4.00	9.00	8.00	10.00	3.00
NUTRICAO	FEMININO	BRANCA	NÃO	HETEROSSEXUAL	SEM COMPANHEIRO	SÓ ESTUDA	NÃO MORA SÓ	5.00	66.00	0.00	11.00	14.00	18.00	7.00
NUTRICAO	FEMININO	BRANCA	NÃO	MINORIAS SEXUAIS	SEM COMPANHEIRO	SÓ ESTUDA	NÃO MORA SÓ	5.00	47.00	0.00	20.00	16.00	23.00	9.00
NUTRICAO	FEMININO	BRANCA	SIM	HETEROSSEXUAL	SEM COMPANHEIRO	SÓ ESTUDA	NÃO MORA SÓ	5.00	42.00	0.00	16.00	19.00	18.00	13.00
NUTRICAO	FEMININO	NÃO BRANCA	SIM	HETEROSSEXUAL	COM COMPANHEIRO	SÓ ESTUDA	NÃO MORA SÓ	5.00	60.00	18.00	12.00	13.00	18.00	10.00
NUTRICAO	FEMININO	NÃO BRANCA	SIM	HETEROSSEXUAL	COM COMPANHEIRO	SÓ ESTUDA	NÃO MORA SÓ	5.00	47.00	21.00				7.00
NUTRICAO	FEMININO	BRANCA	SIM	HETEROSSEXUAL	COM COMPANHEIRO	ESTUDA E TRABALHA	NÃO MORA SÓ	5.00	60.00	0.00	11.00	9.00	16.00	11.00
NUTRICAO	FEMININO	NÃO BRANCA	NÃO	HETEROSSEXUAL	SEM COMPANHEIRO	SÓ ESTUDA	NÃO MORA SÓ	5.00	51.00	0.00	12.00	10.00	17.00	12.00
NUTRICAO	FEMININO	NÃO BRANCA	SIM	HETEROSSEXUAL	COM COMPANHEIRO	ESTUDA E TRABALHA	NÃO MORA SÓ	5.00	76.00	0.00				13.00

NUTRICAO	FEMININO	BRANCA	NÃO	HETEROSSEXUAL	SEM COMPANHEIRO	SÓ ESTUDA	NÃO MORA SÓ	5.00	45.00	7.00	21.00	15.00	21.00	6.00
NUTRICAO	FEMININO	NÃO BRANCA	SIM	HETEROSSEXUAL	SEM COMPANHEIRO	SÓ ESTUDA	NÃO MORA SÓ	5.00	41.00	17.00	16.00	10.00	19.00	13.00
NUTRICAO	FEMININO	BRANCA	SIM	HETEROSSEXUAL	COM COMPANHEIRO	SÓ ESTUDA	MORA SÓ	5.00	42.00	1.00	14.00	17.00	15.00	14.00
NUTRICAO	FEMININO	NÃO BRANCA	SIM	HETEROSSEXUAL	SEM COMPANHEIRO	SÓ ESTUDA	MORA SÓ	5.00	50.00	0.00	18.00	9.00	14.00	12.00
NUTRICAO	FEMININO	NÃO BRANCA	SIM	HETEROSSEXUAL	COM COMPANHEIRO	SÓ ESTUDA	NÃO MORA SÓ	5.00	61.00	1.00	24.00	22.00	23.00	16.00
NUTRICAO	FEMININO	NÃO BRANCA	SIM	HETEROSSEXUAL	COM COMPANHEIRO	SÓ ESTUDA	NÃO MORA SÓ	5.00	67.00	0.00	11.00	16.00	16.00	14.00
NUTRICAO	FEMININO	NÃO BRANCA	SIM	HETEROSSEXUAL	SEM COMPANHEIRO	ESTUDA E TRABALHA	NÃO MORA SÓ	5.00	44.00	4.00	19.00	21.00	20.00	11.00
NUTRICAO	FEMININO	BRANCA	SIM	HETEROSSEXUAL	SEM COMPANHEIRO	SÓ ESTUDA	NÃO MORA SÓ	5.00	68.00	0.00	19.00	17.00	18.00	8.00
NUTRICAO	FEMININO	NÃO BRANCA	SIM	MINORIAS SEXUAIS	SEM COMPANHEIRO	SÓ ESTUDA	NÃO MORA SÓ	3.00	43.00	0.00	10.00	8.00	10.00	12.00
NUTRICAO	FEMININO	NÃO BRANCA	SIM	HETEROSSEXUAL	SEM COMPANHEIRO	ESTUDA E TRABALHA	NÃO MORA SÓ	3.00	55.00	0.00	7.00	10.00	10.00	11.00
NUTRICAO	FEMININO	BRANCA	SIM	HETEROSSEXUAL	SEM COMPANHEIRO	SÓ ESTUDA	NÃO MORA SÓ	3.00	62.00	19.00	14.00	12.00	23.00	13.00
NUTRICAO	FEMININO	NÃO BRANCA	SIM		COM COMPANHEIRO	SÓ ESTUDA	NÃO MORA SÓ	3.00	40.00	8.00	16.00	21.00	23.00	18.00
NUTRICAO	FEMININO	NÃO BRANCA	SIM	HETEROSSEXUAL	SEM COMPANHEIRO	ESTUDA E TRABALHA	NÃO MORA SÓ	3.00	39.00	1.00	28.00	27.00	27.00	13.00
NUTRICAO	FEMININO	NÃO BRANCA	SIM	HETEROSSEXUAL	SEM COMPANHEIRO	SÓ ESTUDA	NÃO MORA SÓ	3.00	70.00	17.00	11.00	12.00	16.00	12.00
NUTRICAO	FEMININO	NÃO BRANCA	SIM	HETEROSSEXUAL	COM COMPANHEIRO	SÓ ESTUDA	NÃO MORA SÓ	3.00	66.00	1.00	19.00	19.00	19.00	10.00
PSICOLOGIA	FEMININO	NÃO BRANCA	NÃO	MINORIAS SEXUAIS	SEM COMPANHEIRO	SÓ ESTUDA	NÃO MORA SÓ	5.00	48.00	1.00	16.00	12.00	13.00	11.00
PSICOLOGIA	FEMININO	BRANCA	SIM	HETEROSSEXUAL			NÃO MORA SÓ	5.00	56.00	0.00	9.00	10.00	19.00	13.00
PSICOLOGIA	FEMININO	NÃO BRANCA	NÃO	MINORIAS SEXUAIS	SEM COMPANHEIRO	SÓ ESTUDA	NÃO MORA SÓ	5.00	39.00	0.00	27.00	23.00	23.00	5.00
PSICOLOGIA	FEMININO	NÃO BRANCA	NÃO	HETEROSSEXUAL	SEM COMPANHEIRO	ESTUDA E TRABALHA	MORA SÓ	5.00	45.00	1.00	14.00	21.00	18.00	17.00
PSICOLOGIA	FEMININO	NÃO BRANCA	NÃO	HETEROSSEXUAL	COM COMPANHEIRO	ESTUDA E TRABALHA	NÃO MORA SÓ	5.00	69.00	0.00	12.00	15.00	17.00	11.00
PSICOLOGIA	FEMININO	NÃO BRANCA	SIM	MINORIAS SEXUAIS	SEM COMPANHEIRO	ESTUDA E TRABALHA	NÃO MORA SÓ	5.00	45.00	0.00	15.00	24.00	24.00	14.00

PSICOLOGIA	FEMININO	BRANCA	SIM	MINORIAS SEXUAIS	SEM COMPANHEIRO	SÓ ESTUDA	NÃO MORA SÓ	5.00	60.00	19.00	25.00	24.00	26.00	12.00
PSICOLOGIA	MASCULINO	NÃO BRANCA	NÃO	MINORIAS SEXUAIS	COM COMPANHEIRO	ESTUDA E TRABALHA	NÃO MORA SÓ	5.00	44.00	19.00	12.00	10.00	15.00	10.00
PSICOLOGIA	FEMININO	NÃO BRANCA	NÃO	MINORIAS SEXUAIS	SEM COMPANHEIRO	ESTUDA E TRABALHA	NÃO MORA SÓ	5.00	48.00	5.00	12.00	9.00	12.00	7.00
PSICOLOGIA	MASCULINO	NÃO BRANCA	NÃO	HETEROSSEXUAL	SEM COMPANHEIRO	SÓ ESTUDA	NÃO MORA SÓ	5.00	59.00	0.00	16.00	19.00	26.00	14.00
PSICOLOGIA	FEMININO	NÃO BRANCA	NÃO	MINORIAS SEXUAIS	SEM COMPANHEIRO	SÓ ESTUDA	NÃO MORA SÓ	5.00	54.00	19.00	23.00	8.00	20.00	11.00
PSICOLOGIA	FEMININO	NÃO BRANCA	SIM	HETEROSSEXUAL	SEM COMPANHEIRO	SÓ ESTUDA	NÃO MORA SÓ	5.00	29.00	0.00	26.00	19.00	26.00	16.00
PSICOLOGIA	FEMININO	BRANCA	SIM	HETEROSSEXUAL	SEM COMPANHEIRO	SÓ ESTUDA	NÃO MORA SÓ	5.00	68.00	0.00	11.00	13.00	19.00	8.00
PSICOLOGIA	MASCULINO	NÃO BRANCA	SIM	MINORIAS SEXUAIS	SEM COMPANHEIRO	ESTUDA E TRABALHA	NÃO MORA SÓ	5.00	51.00	17.00	20.00	21.00	22.00	8.00
PSICOLOGIA	FEMININO	NÃO BRANCA	SIM	HETEROSSEXUAL	SEM COMPANHEIRO	SÓ ESTUDA	NÃO MORA SÓ	5.00	55.00	0.00	14.00	17.00	19.00	8.00
PSICOLOGIA	FEMININO	NÃO BRANCA	NÃO	HETEROSSEXUAL	SEM COMPANHEIRO	SÓ ESTUDA	NÃO MORA SÓ	5.00	60.00	6.00	15.00	8.00	13.00	11.00
PSICOLOGIA	MASCULINO	BRANCA	NÃO	MINORIAS SEXUAIS	SEM COMPANHEIRO	SÓ ESTUDA	MORA SÓ	5.00	30.00	19.00	28.00	20.00	26.00	16.00
PSICOLOGIA	FEMININO	BRANCA	NÃO	MINORIAS SEXUAIS	SEM COMPANHEIRO	SÓ ESTUDA	NÃO MORA SÓ	5.00	37.00	12.00	19.00	23.00	26.00	2.00
PSICOLOGIA	FEMININO	BRANCA	NÃO	MINORIAS SEXUAIS	SEM COMPANHEIRO	SÓ ESTUDA	NÃO MORA SÓ	5.00	48.00	4.00	12.00	11.00	19.00	9.00
PSICOLOGIA	FEMININO	NÃO BRANCA	SIM	MINORIAS SEXUAIS	COM COMPANHEIRO	ESTUDA E TRABALHA	NÃO MORA SÓ	5.00	48.00	23.00	22.00	25.00	28.00	12.00
PSICOLOGIA	FEMININO	BRANCA	NÃO	MINORIAS SEXUAIS	SEM COMPANHEIRO	SÓ ESTUDA	NÃO MORA SÓ	5.00	55.00	4.00	16.00	14.00	16.00	4.00
MEDICINA	FEMININO	NÃO BRANCA	SIM	HETEROSSEXUAL	SEM COMPANHEIRO	SÓ ESTUDA	NÃO MORA SÓ	1.00	67.00	0.00	7.00	9.00	8.00	12.00
MEDICINA	MASCULINO	NÃO BRANCA	SIM	MINORIAS SEXUAIS	SEM COMPANHEIRO	ESTUDA E TRABALHA	MORA SÓ	1.00	66.00	0.00	11.00	11.00	24.00	15.00
MEDICINA	MASCULINO	NÃO BRANCA	NÃO	HETEROSSEXUAL	SEM COMPANHEIRO	SÓ ESTUDA	NÃO MORA SÓ	1.00	47.00	0.00	14.00	14.00	15.00	8.00
MEDICINA	FEMININO	NÃO BRANCA	SIM	MINORIAS SEXUAIS	SEM COMPANHEIRO	ESTUDA E TRABALHA	NÃO MORA SÓ	1.00	28.00	19.00	14.00	16.00	17.00	16.00
MEDICINA	MASCULINO	BRANCA	SIM	MINORIAS SEXUAIS	SEM COMPANHEIRO	SÓ ESTUDA	NÃO MORA SÓ	1.00	43.00	1.00	27.00	10.00	18.00	13.00
MEDICINA	MASCULINO	NÃO BRANCA	NÃO	MINORIAS SEXUAIS	SEM COMPANHEIRO	SÓ ESTUDA	NÃO MORA SÓ	1.00	56.00	0.00	9.00	10.00	9.00	10.00

MEDICINA	MASCULINO	BRANCA	SIM	HETEROSSEXUAL	SEM COMPANHEIRO	SÓ ESTUDA	NÃO MORA SÓ	1.00	56.00	0.00	10.00	10.00	17.00	11.00
MEDICINA	FEMININO	BRANCA	SIM	HETEROSSEXUAL	SEM COMPANHEIRO	SÓ ESTUDA	NÃO MORA SÓ	1.00	58.00	0.00	11.00	8.00	19.00	14.00
MEDICINA	MASCULINO	NÃO BRANCA	SIM	HETEROSSEXUAL	SEM COMPANHEIRO	SÓ ESTUDA	NÃO MORA SÓ	1.00	69.00	0.00	8.00	10.00	10.00	10.00
MEDICINA	MASCULINO	NÃO BRANCA	SIM	HETEROSSEXUAL	SEM COMPANHEIRO	SÓ ESTUDA	NÃO MORA SÓ	1.00	66.00	0.00	13.00	7.00	12.00	7.00
MEDICINA	MASCULINO	NÃO BRANCA	SIM	HETEROSSEXUAL	SEM COMPANHEIRO	SÓ ESTUDA	NÃO MORA SÓ	1.00	34.00	0.00	24.00	17.00	22.00	3.00
MEDICINA	FEMININO	NÃO BRANCA	NÃO	HETEROSSEXUAL	SEM COMPANHEIRO	SÓ ESTUDA	MORA SÓ	1.00	47.00	0.00	14.00	8.00	11.00	13.00
MEDICINA	MASCULINO	BRANCA	NÃO	HETEROSSEXUAL	COM COMPANHEIRO	SÓ ESTUDA	NÃO MORA SÓ	1.00	87.00	0.00	8.00	8.00	10.00	5.00
MEDICINA	MASCULINO	NÃO BRANCA	SIM	HETEROSSEXUAL	COM COMPANHEIRO	ESTUDA E TRABALHA	NÃO MORA SÓ	1.00	36.00	7.00	12.00	13.00	24.00	15.00
MEDICINA	MASCULINO	NÃO BRANCA	SIM	HETEROSSEXUAL	SEM COMPANHEIRO	ESTUDA E TRABALHA	MORA SÓ	1.00	43.00	1.00	13.00	15.00	13.00	14.00
MEDICINA	MASCULINO	NÃO BRANCA	SIM	HETEROSSEXUAL	COM COMPANHEIRO	SÓ ESTUDA	NÃO MORA SÓ	1.00	43.00	1.00	11.00	13.00	14.00	12.00
MEDICINA	FEMININO	NÃO BRANCA	NÃO	HETEROSSEXUAL	SEM COMPANHEIRO	SÓ ESTUDA	NÃO MORA SÓ	1.00	48.00	19.00	15.00	17.00	21.00	11.00
MEDICINA	MASCULINO	BRANCA	SIM	HETEROSSEXUAL	SEM COMPANHEIRO	SÓ ESTUDA	MORA SÓ	1.00	47.00	2.00	13.00	8.00	18.00	9.00
MEDICINA	MASCULINO	BRANCA	SIM	MINORIAS SEXUAIS	COM COMPANHEIRO	SÓ ESTUDA	MORA SÓ	1.00	41.00	0.00	17.00	15.00	20.00	18.00
MEDICINA	FEMININO	NÃO BRANCA	SIM	HETEROSSEXUAL	COM COMPANHEIRO	ESTUDA E TRABALHA	NÃO MORA SÓ	1.00	73.00	0.00	6.00	7.00	10.00	8.00
MEDICINA	MASCULINO	NÃO BRANCA	SIM	HETEROSSEXUAL	SEM COMPANHEIRO	SÓ ESTUDA	NÃO MORA SÓ	1.00	67.00	0.00	7.00	7.00	7.00	0.00
MEDICINA	FEMININO	BRANCA	NÃO	HETEROSSEXUAL	SEM COMPANHEIRO	SÓ ESTUDA	NÃO MORA SÓ	1.00	56.00	0.00	8.00	10.00	10.00	6.00
MEDICINA	MASCULINO	NÃO BRANCA	NÃO	HETEROSSEXUAL	COM COMPANHEIRO	SÓ ESTUDA	MORA SÓ	1.00	24.00	7.00	21.00	14.00	25.00	16.00
MEDICINA	MASCULINO	BRANCA	SIM	HETEROSSEXUAL	COM COMPANHEIRO	ESTUDA E TRABALHA	NÃO MORA SÓ	1.00	35.00	3.00	28.00	28.00	28.00	18.00
MEDICINA	FEMININO	BRANCA	SIM	HETEROSSEXUAL	SEM COMPANHEIRO	ESTUDA E TRABALHA	MORA SÓ	1.00	34.00	0.00	18.00	19.00	20.00	9.00
MEDICINA	FEMININO	BRANCA	SIM	HETEROSSEXUAL	COM COMPANHEIRO	SÓ ESTUDA	MORA SÓ	1.00	59.00	0.00	9.00	10.00	14.00	6.00
MEDICINA	FEMININO	NÃO BRANCA	SIM	HETEROSSEXUAL	SEM COMPANHEIRO	ESTUDA E TRABALHA	MORA SÓ	1.00	55.00	4.00	9.00	10.00	16.00	3.00

MEDICINA	MASCULINO	NÃO BRANCA	NÃO	MINORIAS SEXUAIS	SEM COMPANHEIRO	ESTUDA E TRABALHA	NÃO MORA SÓ	1.00	56.00	0.00	8.00	9.00	15.00	15.00
MEDICINA	FEMININO	NÃO BRANCA	SIM	HETEROSSEXUAL	COM COMPANHEIRO	SÓ ESTUDA	MORA SÓ	1.00	58.00	0.00	12.00	7.00	13.00	10.00
MEDICINA	FEMININO	BRANCA	NÃO	HETEROSSEXUAL	COM COMPANHEIRO	SÓ ESTUDA	NÃO MORA SÓ	1.00	49.00	17.00	19.00	16.00	24.00	9.00
MEDICINA	FEMININO	NÃO BRANCA	NÃO	HETEROSSEXUAL	SEM COMPANHEIRO	SÓ ESTUDA	NÃO MORA SÓ	1.00	58.00	0.00	13.00	11.00	17.00	10.00
MEDICINA	MASCULINO	NÃO BRANCA	SIM	HETEROSSEXUAL	SEM COMPANHEIRO	SÓ ESTUDA	NÃO MORA SÓ	1.00	56.00	0.00	11.00	8.00	13.00	7.00
MEDICINA	FEMININO	BRANCA	SIM	HETEROSSEXUAL	SEM COMPANHEIRO	SÓ ESTUDA	MORA SÓ	1.00	48.00	0.00	8.00	9.00	12.00	8.00
MEDICINA	FEMININO	BRANCA	NÃO	HETEROSSEXUAL	COM COMPANHEIRO	SÓ ESTUDA	NÃO MORA SÓ	1.00	48.00	33.00	18.00	18.00	16.00	13.00
MEDICINA	FEMININO	BRANCA	NÃO	MINORIAS SEXUAIS	SEM COMPANHEIRO	SÓ ESTUDA	NÃO MORA SÓ	1.00	52.00	0.00	11.00	7.00	15.00	12.00
MEDICINA	FEMININO	BRANCA	SIM	MINORIAS SEXUAIS	SEM COMPANHEIRO	SÓ ESTUDA	MORA SÓ	1.00	19.00	5.00	19.00	16.00	17.00	12.00
MEDICINA	MASCULINO	BRANCA	SIM	HETEROSSEXUAL	SEM COMPANHEIRO	SÓ ESTUDA	NÃO MORA SÓ	1.00	39.00	0.00	14.00	23.00	20.00	12.00
MEDICINA	FEMININO	BRANCA	SIM	HETEROSSEXUAL	COM COMPANHEIRO	SÓ ESTUDA	NÃO MORA SÓ	1.00	53.00	0.00	8.00	11.00	13.00	5.00
MEDICINA	FEMININO	NÃO BRANCA	SIM	HETEROSSEXUAL	SEM COMPANHEIRO	SÓ ESTUDA	NÃO MORA SÓ	5.00	40.00	23.00	27.00	14.00	25.00	18.00
MEDICINA	MASCULINO	NÃO BRANCA	SIM		COM COMPANHEIRO	ESTUDA E TRABALHA	NÃO MORA SÓ	5.00	86.00	0.00	11.00	10.00	12.00	11.00
MEDICINA	MASCULINO	BRANCA	NÃO	HETEROSSEXUAL	COM COMPANHEIRO	SÓ ESTUDA	NÃO MORA SÓ	3.00	64.00	9.00	15.00	10.00	10.00	18.00
MEDICINA	MASCULINO	NÃO BRANCA	SIM	HETEROSSEXUAL	COM COMPANHEIRO		NÃO MORA SÓ		50.00	1.00	20.00	17.00	19.00	3.00
MEDICINA	MASCULINO	NÃO BRANCA	NÃO	HETEROSSEXUAL	SEM COMPANHEIRO	SÓ ESTUDA	NÃO MORA SÓ	3.00	69.00	0.00	8.00	8.00	11.00	8.00
MEDICINA	MASCULINO	NÃO BRANCA	NÃO	HETEROSSEXUAL	SEM COMPANHEIRO	SÓ ESTUDA	NÃO MORA SÓ	3.00	60.00	0.00	9.00	8.00	8.00	15.00
MEDICINA	FEMININO	BRANCA	NÃO	HETEROSSEXUAL	SEM COMPANHEIRO	SÓ ESTUDA	MORA SÓ	3.00	41.00	0.00	10.00	9.00	12.00	13.00
MEDICINA	MASCULINO	BRANCA	NÃO	MINORIAS SEXUAIS	SEM COMPANHEIRO	SÓ ESTUDA	MORA SÓ	3.00	62.00	0.00	10.00	9.00	16.00	13.00
MEDICINA	MASCULINO	BRANCA	SIM	HETEROSSEXUAL	COM COMPANHEIRO	SÓ ESTUDA	NÃO MORA SÓ	3.00	57.00	0.00	12.00	8.00	13.00	10.00
MEDICINA	MASCULINO	NÃO BRANCA	NÃO	MINORIAS SEXUAIS	SEM COMPANHEIRO		NÃO MORA SÓ	3.00	40.00	0.00	15.00	8.00	18.00	13.00

MEDICINA	FEMININO	NÃO BRANCA	NÃO	MINORIAS SEXUAIS	SEM COMPANHEIRO	SÓ ESTUDA	NÃO MORA SÓ	3.00	63.00	0.00	9.00	10.00	11.00	18.00
MEDICINA	MASCULINO	BRANCA	SIM	MINORIAS SEXUAIS	SEM COMPANHEIRO	SÓ ESTUDA	MORA SÓ	3.00	83.00	0.00	12.00	11.00	18.00	5.00
MEDICINA	FEMININO	BRANCA	NÃO	HETEROSSEXUAL	SEM COMPANHEIRO	SÓ ESTUDA	NÃO MORA SÓ	3.00	69.00	3.00	14.00	7.00	16.00	7.00
MEDICINA	MASCULINO	NÃO BRANCA	NÃO	HETEROSSEXUAL	COM COMPANHEIRO	SÓ ESTUDA	NÃO MORA SÓ	3.00	61.00	0.00	9.00	7.00	13.00	6.00
MEDICINA	MASCULINO	NÃO BRANCA	SIM	HETEROSSEXUAL	COM COMPANHEIRO	ESTUDA E TRABALHA	NÃO MORA SÓ	3.00	76.00	0.00	9.00	8.00	17.00	9.00
MEDICINA	MASCULINO	BRANCA	SIM	HETEROSSEXUAL	SEM COMPANHEIRO	SÓ ESTUDA	MORA SÓ	3.00	42.00	0.00	11.00	8.00	14.00	14.00
MEDICINA	MASCULINO	BRANCA	NÃO	HETEROSSEXUAL	COM COMPANHEIRO	SÓ ESTUDA	NÃO MORA SÓ	3.00	53.00	0.00	14.00	8.00	16.00	11.00
MEDICINA	MASCULINO	NÃO BRANCA	SIM	HETEROSSEXUAL	COM COMPANHEIRO	SÓ ESTUDA	NÃO MORA SÓ	3.00	90.00	0.00	9.00	7.00	13.00	14.00
MEDICINA	FEMININO	BRANCA	NÃO	MINORIAS SEXUAIS	SEM COMPANHEIRO	SÓ ESTUDA	NÃO MORA SÓ	3.00	45.00	19.00	27.00	24.00	26.00	4.00
MEDICINA	FEMININO	NÃO BRANCA	NÃO	HETEROSSEXUAL	COM COMPANHEIRO	SÓ ESTUDA	NÃO MORA SÓ	3.00	65.00	0.00	15.00	14.00	24.00	10.00
MEDICINA	FEMININO	BRANCA	SIM	HETEROSSEXUAL	SEM COMPANHEIRO	SÓ ESTUDA	MORA SÓ	3.00	57.00	0.00	8.00	10.00	10.00	11.00
MEDICINA	MASCULINO	BRANCA	NÃO	MINORIAS SEXUAIS	SEM COMPANHEIRO	SÓ ESTUDA	MORA SÓ	3.00	49.00	0.00	16.00	14.00	14.00	11.00
MEDICINA	MASCULINO	NÃO BRANCA	NÃO	MINORIAS SEXUAIS	SEM COMPANHEIRO	SÓ ESTUDA	MORA SÓ	3.00	40.00	4.00	19.00	16.00	18.00	15.00
MEDICINA	FEMININO	NÃO BRANCA	SIM	HETEROSSEXUAL	SEM COMPANHEIRO	SÓ ESTUDA	MORA SÓ	3.00	85.00	0.00	7.00	7.00	10.00	10.00
MEDICINA	FEMININO	BRANCA	SIM	HETEROSSEXUAL	SEM COMPANHEIRO	SÓ ESTUDA	MORA SÓ	3.00	34.00	18.00	20.00	24.00	24.00	14.00
MEDICINA	FEMININO	BRANCA	SIM	HETEROSSEXUAL	SEM COMPANHEIRO	SÓ ESTUDA	NÃO MORA SÓ	3.00	40.00	0.00	14.00	15.00	22.00	16.00
MEDICINA	FEMININO	NÃO BRANCA	SIM	HETEROSSEXUAL	SEM COMPANHEIRO	SÓ ESTUDA	NÃO MORA SÓ	3.00	54.00	2.00	8.00	8.00	13.00	9.00
NUTRICAO	FEMININO	NÃO BRANCA	NÃO	HETEROSSEXUAL	SEM COMPANHEIRO	SÓ ESTUDA	NÃO MORA SÓ	3.00	57.00	7.00	12.00	9.00	17.00	9.00
NUTRICAO	FEMININO	NÃO BRANCA	SIM	HETEROSSEXUAL	SEM COMPANHEIRO	SÓ ESTUDA	NÃO MORA SÓ	3.00	31.00	0.00	21.00	12.00	23.00	12.00
NUTRICAO	FEMININO	NÃO BRANCA	SIM	HETEROSSEXUAL	SEM COMPANHEIRO	SÓ ESTUDA	MORA SÓ	3.00	48.00	0.00	7.00	9.00	14.00	12.00
NUTRICAO	FEMININO	NÃO BRANCA	SIM	HETEROSSEXUAL	COM COMPANHEIRO	SÓ ESTUDA	NÃO MORA SÓ	3.00	48.00	0.00	10.00	13.00	17.00	6.00

NUTRICAO	MASCULINO	NÃO BRANCA	NÃO	HETEROSSEXUAL	COM COMPANHEIRO	ESTUDA E TRABALHA	NÃO MORA SÓ	3.00	60.00	0.00	9.00	9.00	13.00	
NUTRICAO	FEMININO	BRANCA	NÃO	HETEROSSEXUAL	SEM COMPANHEIRO	SÓ ESTUDA	NÃO MORA SÓ	3.00	60.00	7.00	15.00	25.00	23.00	3.00
NUTRICAO	FEMININO	NÃO BRANCA	SIM	HETEROSSEXUAL	SEM COMPANHEIRO	SÓ ESTUDA	NÃO MORA SÓ	3.00	38.00	19.00	23.00	22.00	26.00	7.00
NUTRICAO	FEMININO	NÃO BRANCA	SIM	HETEROSSEXUAL	COM COMPANHEIRO	ESTUDA E TRABALHA	NÃO MORA SÓ	3.00	55.00	0.00	10.00	9.00	16.00	6.00
NUTRICAO	FEMININO	NÃO BRANCA	SIM	HETEROSSEXUAL			NÃO MORA SÓ	3.00	60.00	0.00	10.00	8.00	9.00	4.00
NUTRICAO	FEMININO	BRANCA	NÃO	HETEROSSEXUAL	COM COMPANHEIRO	SÓ ESTUDA	NÃO MORA SÓ	3.00	41.00	1.00	17.00	15.00	14.00	11.00
NUTRICAO	FEMININO	NÃO BRANCA	SIM	HETEROSSEXUAL	SEM COMPANHEIRO	SÓ ESTUDA	NÃO MORA SÓ	3.00	53.00	0.00	8.00	11.00	11.00	7.00
NUTRICAO	MASCULINO	NÃO BRANCA	SIM	HETEROSSEXUAL	SEM COMPANHEIRO	SÓ ESTUDA	NÃO MORA SÓ	3.00	68.00	0.00			8.00	14.00
NUTRICAO	FEMININO	NÃO BRANCA	SIM	HETEROSSEXUAL	SEM COMPANHEIRO	SÓ ESTUDA	MORA SÓ	3.00	23.00	1.00	21.00	18.00	27.00	5.00
NUTRICAO	FEMININO	NÃO BRANCA	SIM	HETEROSSEXUAL	COM COMPANHEIRO	SÓ ESTUDA	NÃO MORA SÓ	3.00	60.00	0.00	8.00	8.00	13.00	10.00
NUTRICAO	FEMININO	BRANCA	SIM	HETEROSSEXUAL	SEM COMPANHEIRO	SÓ ESTUDA	NÃO MORA SÓ	3.00	43.00	4.00	17.00	12.00	18.00	9.00
NUTRICAO	FEMININO	NÃO BRANCA	SIM	HETEROSSEXUAL	SEM COMPANHEIRO	SÓ ESTUDA	MORA SÓ	3.00	48.00	1.00	26.00	20.00	24.00	12.00
PSICOLOGIA	FEMININO	BRANCA	SIM	HETEROSSEXUAL	SEM COMPANHEIRO	SÓ ESTUDA	NÃO MORA SÓ	0.00	57.00	0.00	13.00	13.00	14.00	7.00
PSICOLOGIA	FEMININO	BRANCA	SIM	HETEROSSEXUAL	SEM COMPANHEIRO	ESTUDA E TRABALHA	NÃO MORA SÓ	0.00	40.00	0.00	13.00	8.00	15.00	9.00
PSICOLOGIA	FEMININO	NÃO BRANCA	NÃO	MINORIAS SEXUAIS		SÓ ESTUDA	NÃO MORA SÓ	0.00	40.00	2.00	14.00	9.00	16.00	12.00
PSICOLOGIA	FEMININO	BRANCA	NÃO	MINORIAS SEXUAIS	SEM COMPANHEIRO	ESTUDA E TRABALHA	NÃO MORA SÓ	0.00	50.00	8.00	14.00	14.00	16.00	13.00
PSICOLOGIA	FEMININO	BRANCA	SIM	HETEROSSEXUAL	SEM COMPANHEIRO	SÓ ESTUDA	NÃO MORA SÓ	5.00	56.00	0.00	13.00	9.00	13.00	10.00
PSICOLOGIA	FEMININO	BRANCA	NÃO	MINORIAS SEXUAIS	SEM COMPANHEIRO	SÓ ESTUDA	NÃO MORA SÓ	0.00	40.00	13.00	26.00	21.00	15.00	11.00
PSICOLOGIA	MASCULINO	BRANCA	NÃO	HETEROSSEXUAL	COM COMPANHEIRO	ESTUDA E TRABALHA	NÃO MORA SÓ	0.00	45.00	0.00	11.00	13.00	16.00	6.00
PSICOLOGIA	MASCULINO	NÃO BRANCA	NÃO	MINORIAS SEXUAIS	SEM COMPANHEIRO	SÓ ESTUDA	NÃO MORA SÓ	0.00	70.00	0.00	8.00	9.00	7.00	5.00
PSICOLOGIA	FEMININO	BRANCA	NÃO	MINORIAS SEXUAIS	COM COMPANHEIRO	SÓ ESTUDA	NÃO MORA SÓ	0.00	44.00	3.00	19.00	17.00	21.00	12.00

PSICOLOGIA	FEMININO	BRANCA	SIM	HETEROSSEXUAL	SEM COMPANHEIRO	SÓ ESTUDA	NÃO MORA SÓ	0.00	50.00	0.00	10.00	13.00	13.00	13.00
PSICOLOGIA	FEMININO	NÃO BRANCA	NÃO	HETEROSSEXUAL	SEM COMPANHEIRO	SÓ ESTUDA	NÃO MORA SÓ	0.00	64.00	0.00	11.00	14.00	15.00	7.00
PSICOLOGIA	MASCULINO	NÃO BRANCA	SIM	HETEROSSEXUAL	COM COMPANHEIRO	ESTUDA E TRABALHA	NÃO MORA SÓ	0.00	57.00	0.00	13.00	10.00	16.00	7.00
PSICOLOGIA	MASCULINO	NÃO BRANCA	NÃO	HETEROSSEXUAL	SEM COMPANHEIRO	SÓ ESTUDA	NÃO MORA SÓ	0.00	41.00	0.00	15.00	7.00	18.00	
PSICOLOGIA	FEMININO	BRANCA	SIM	HETEROSSEXUAL	SEM COMPANHEIRO	SÓ ESTUDA	NÃO MORA SÓ	0.00	65.00	0.00	8.00	10.00	14.00	10.00
PSICOLOGIA	FEMININO	NÃO BRANCA	NÃO	MINORIAS SEXUAIS	SEM COMPANHEIRO	SÓ ESTUDA	NÃO MORA SÓ	0.00	53.00	4.00	13.00	11.00	16.00	9.00
PSICOLOGIA	MASCULINO	BRANCA	NÃO	HETEROSSEXUAL	SEM COMPANHEIRO	SÓ ESTUDA	NÃO MORA SÓ	0.00	68.00	0.00	12.00	9.00	14.00	6.00
PSICOLOGIA	FEMININO	NÃO BRANCA	NÃO	MINORIAS SEXUAIS	SEM COMPANHEIRO	SÓ ESTUDA	NÃO MORA SÓ	0.00	53.00	9.00	15.00	12.00	16.00	7.00
PSICOLOGIA	FEMININO	BRANCA	SIM	HETEROSSEXUAL	SEM COMPANHEIRO	ESTUDA E TRABALHA	NÃO MORA SÓ	0.00	61.00	7.00	16.00	10.00	9.00	10.00
PSICOLOGIA	FEMININO	BRANCA	NÃO	HETEROSSEXUAL	SEM COMPANHEIRO	SÓ ESTUDA	MORA SÓ	0.00	78.00	0.00	7.00	10.00	7.00	5.00
PSICOLOGIA	FEMININO	BRANCA	SIM	MINORIAS SEXUAIS	SEM COMPANHEIRO	SÓ ESTUDA	NÃO MORA SÓ	0.00	38.00	19.00	24.00	24.00	26.00	11.00
PSICOLOGIA	MASCULINO	BRANCA	NÃO	HETEROSSEXUAL	SEM COMPANHEIRO	ESTUDA E TRABALHA	NÃO MORA SÓ	0.00	56.00	17.00	27.00	21.00	23.00	
PSICOLOGIA		BRANCA	NÃO		SEM COMPANHEIRO	SÓ ESTUDA	NÃO MORA SÓ	0.00	61.00	4.00	10.00	13.00	16.00	6.00
PSICOLOGIA	FEMININO	NÃO BRANCA	SIM	MINORIAS SEXUAIS	COM COMPANHEIRO	SÓ ESTUDA	NÃO MORA SÓ	0.00	62.00	4.00	18.00	12.00	21.00	13.00
PSICOLOGIA	MASCULINO	NÃO BRANCA	NÃO	MINORIAS SEXUAIS	SEM COMPANHEIRO	SÓ ESTUDA	MORA SÓ	0.00	29.00	1.00	22.00	27.00	24.00	3.00
PSICOLOGIA	FEMININO	BRANCA	NÃO	MINORIAS SEXUAIS	SEM COMPANHEIRO	SÓ ESTUDA	NÃO MORA SÓ	0.00	56.00	0.00	11.00	17.00	16.00	13.00
PSICOLOGIA	FEMININO	NÃO BRANCA	SIM	MINORIAS SEXUAIS	SEM COMPANHEIRO	SÓ ESTUDA	NÃO MORA SÓ	0.00	53.00	19.00	21.00	22.00	20.00	12.00
PSICOLOGIA	FEMININO	NÃO BRANCA	SIM	HETEROSSEXUAL	SEM COMPANHEIRO	SÓ ESTUDA	MORA SÓ	0.00	54.00	0.00	16.00	14.00	13.00	9.00
PSICOLOGIA	FEMININO	NÃO BRANCA	SIM	HETEROSSEXUAL	SEM COMPANHEIRO	SÓ ESTUDA	NÃO MORA SÓ	0.00	35.00	0.00	18.00	13.00	21.00	12.00
PSICOLOGIA	FEMININO	NÃO BRANCA	SIM	HETEROSSEXUAL	SEM COMPANHEIRO	SÓ ESTUDA	NÃO MORA SÓ	0.00	39.00	0.00	11.00	9.00	16.00	9.00
PSICOLOGIA	MASCULINO	NÃO BRANCA	NÃO	MINORIAS SEXUAIS	SEM COMPANHEIRO	SÓ ESTUDA	MORA SÓ	0.00	37.00	9.00	22.00	18.00	23.00	10.00

ENFERMAGEM	FEMININO	NÃO BRANCA	NÃO	MINORIAS SEXUAIS	COM COMPANHEIRO	ESTUDA E TRABALHA	NÃO MORA SÓ	5.00	42.00	23.00	25.00	15.00	20.00	15.00
ENFERMAGEM	MASCULINO	BRANCA	SIM	MINORIAS SEXUAIS	SEM COMPANHEIRO	SÓ ESTUDA	MORA SÓ	1.00	56.00	5.00	23.00	15.00	19.00	10.00
ENFERMAGEM	FEMININO	NÃO BRANCA	SIM	HETEROSSEXUAL	SEM COMPANHEIRO	SÓ ESTUDA	NÃO MORA SÓ	1.00	56.00	9.00	23.00	19.00	19.00	17.00
ENFERMAGEM	FEMININO	NÃO BRANCA	SIM	HETEROSSEXUAL	COM COMPANHEIRO	SÓ ESTUDA	NÃO MORA SÓ	1.00	42.00	13.00	25.00	21.00	28.00	8.00
ENFERMAGEM	FEMININO	NÃO BRANCA	SIM	MINORIAS SEXUAIS	COM COMPANHEIRO	SÓ ESTUDA	NÃO MORA SÓ	1.00	41.00	5.00	16.00	17.00	24.00	7.00
ENFERMAGEM	FEMININO	BRANCA	NÃO	MINORIAS SEXUAIS	COM COMPANHEIRO	SÓ ESTUDA	NÃO MORA SÓ	1.00	34.00	9.00	25.00	20.00	24.00	11.00
ENFERMAGEM	FEMININO	NÃO BRANCA	SIM	HETEROSSEXUAL	COM COMPANHEIRO	SÓ ESTUDA	NÃO MORA SÓ	5.00	45.00	9.00	27.00	25.00	25.00	11.00
ENFERMAGEM	FEMININO	BRANCA	SIM	MINORIAS SEXUAIS	COM COMPANHEIRO	ESTUDA E TRABALHA	NÃO MORA SÓ	1.00	31.00	23.00	17.00	14.00	21.00	15.00
ENFERMAGEM	FEMININO	NÃO BRANCA	SIM	HETEROSSEXUAL	SEM COMPANHEIRO	SÓ ESTUDA	NÃO MORA SÓ	1.00	59.00	0.00	7.00	10.00	11.00	7.00
ENFERMAGEM	FEMININO	NÃO BRANCA	NÃO	HETEROSSEXUAL	COM COMPANHEIRO	SÓ ESTUDA	NÃO MORA SÓ	1.00	55.00	0.00	21.00	14.00	14.00	2.00
ENFERMAGEM	FEMININO	NÃO BRANCA	SIM	HETEROSSEXUAL	SEM COMPANHEIRO	SÓ ESTUDA	NÃO MORA SÓ	5.00	58.00	0.00	8.00	10.00	14.00	4.00
ENFERMAGEM	FEMININO	NÃO BRANCA	NÃO	HETEROSSEXUAL	SEM COMPANHEIRO	SÓ ESTUDA	NÃO MORA SÓ	1.00	56.00	0.00	24.00	24.00	25.00	13.00
ENFERMAGEM	MASCULINO	NÃO BRANCA	SIM	MINORIAS SEXUAIS	SEM COMPANHEIRO	SÓ ESTUDA	NÃO MORA SÓ	5.00	56.00	0.00	15.00	20.00	20.00	11.00
ENFERMAGEM	FEMININO	BRANCA	SIM	HETEROSSEXUAL	SEM COMPANHEIRO	SÓ ESTUDA	NÃO MORA SÓ	1.00	51.00	0.00	11.00	15.00	15.00	8.00
ENFERMAGEM	FEMININO	NÃO BRANCA	SIM	HETEROSSEXUAL	SEM COMPANHEIRO	SÓ ESTUDA	NÃO MORA SÓ	1.00	63.00	0.00	10.00	8.00	12.00	11.00
ENFERMAGEM	FEMININO	NÃO BRANCA	SIM	HETEROSSEXUAL	SEM COMPANHEIRO	ESTUDA E TRABALHA	NÃO MORA SÓ	1.00	72.00	0.00	7.00	10.00	11.00	9.00
ENFERMAGEM	FEMININO	NÃO BRANCA	SIM	HETEROSSEXUAL	SEM COMPANHEIRO	SÓ ESTUDA	NÃO MORA SÓ	1.00	55.00	0.00	13.00	10.00	13.00	9.00
ENFERMAGEM	MASCULINO	NÃO BRANCA	NÃO	MINORIAS SEXUAIS	SEM COMPANHEIRO	SÓ ESTUDA	MORA SÓ	1.00	48.00	0.00	13.00	7.00	13.00	16.00
ENFERMAGEM	FEMININO	NÃO BRANCA	SIM	HETEROSSEXUAL	SEM COMPANHEIRO	SÓ ESTUDA	NÃO MORA SÓ	1.00	66.00	0.00	20.00	7.00	13.00	10.00
ENFERMAGEM	MASCULINO	BRANCA	NÃO	MINORIAS SEXUAIS	SEM COMPANHEIRO	SÓ ESTUDA	NÃO MORA SÓ	1.00	38.00	0.00	11.00	18.00	22.00	15.00
ENFERMAGEM	FEMININO	BRANCA	SIM	HETEROSSEXUAL	COM COMPANHEIRO	SÓ ESTUDA	NÃO MORA SÓ	1.00	60.00	4.00	11.00	9.00	16.00	2.00

ENFERMAGEM	FEMININO	NÃO BRANCA	SIM	HETEROSSEXUAL	SEM COMPANHEIRO	SÓ ESTUDA	NÃO MORA SÓ	1.00	55.00	0.00	21.00	17.00	20.00	8.00
ENFERMAGEM	FEMININO	NÃO BRANCA	NÃO	MINORIAS SEXUAIS	COM COMPANHEIRO	SÓ ESTUDA	NÃO MORA SÓ	5.00	60.00	33.00	14.00	17.00	19.00	18.00
ENFERMAGEM	FEMININO	BRANCA	SIM	HETEROSSEXUAL	COM COMPANHEIRO	SÓ ESTUDA	MORA SÓ	5.00	31.00	3.00	28.00	19.00	28.00	14.00
ENFERMAGEM	FEMININO	BRANCA	NÃO	MINORIAS SEXUAIS	COM COMPANHEIRO	SÓ ESTUDA	NÃO MORA SÓ	5.00	50.00	19.00	23.00	14.00	19.00	10.00
ENFERMAGEM	FEMININO	NÃO BRANCA	SIM	HETEROSSEXUAL	SEM COMPANHEIRO	SÓ ESTUDA	NÃO MORA SÓ	5.00	41.00	0.00	15.00	12.00	17.00	14.00
ENFERMAGEM	FEMININO	NÃO BRANCA	NÃO	MINORIAS SEXUAIS	SEM COMPANHEIRO	SÓ ESTUDA	MORA SÓ	5.00	54.00	7.00	22.00	17.00	19.00	9.00
ENFERMAGEM	FEMININO	NÃO BRANCA	NÃO	MINORIAS SEXUAIS	SEM COMPANHEIRO	SÓ ESTUDA	NÃO MORA SÓ	5.00	53.00	1.00	18.00	15.00	21.00	10.00
ENFERMAGEM	FEMININO	NÃO BRANCA	SIM	HETEROSSEXUAL	SEM COMPANHEIRO	SÓ ESTUDA	NÃO MORA SÓ	5.00	39.00	0.00	13.00	9.00	22.00	13.00
ENFERMAGEM	FEMININO	NÃO BRANCA	SIM	HETEROSSEXUAL	COM COMPANHEIRO	SÓ ESTUDA	NÃO MORA SÓ	5.00	51.00	0.00	15.00	21.00	24.00	18.00
ENFERMAGEM	MASCULINO	NÃO BRANCA	NÃO	MINORIAS SEXUAIS	SEM COMPANHEIRO	SÓ ESTUDA	MORA SÓ	5.00	52.00	0.00	10.00	7.00	13.00	11.00
ENFERMAGEM	MASCULINO	NÃO BRANCA	SIM	HETEROSSEXUAL	COM COMPANHEIRO	SÓ ESTUDA	NÃO MORA SÓ	5.00	48.00	0.00	9.00	8.00	15.00	5.00
ENFERMAGEM	FEMININO	BRANCA	SIM	HETEROSSEXUAL	SEM COMPANHEIRO	SÓ ESTUDA	NÃO MORA SÓ	3.00	37.00	19.00	26.00	17.00	22.00	12.00
ENFERMAGEM	FEMININO	NÃO BRANCA	SIM	MINORIAS SEXUAIS	SEM COMPANHEIRO	SÓ ESTUDA	MORA SÓ	3.00	59.00	7.00	14.00	14.00	17.00	15.00
ENFERMAGEM	FEMININO	NÃO BRANCA	NÃO	MINORIAS SEXUAIS	COM COMPANHEIRO	SÓ ESTUDA	NÃO MORA SÓ	3.00	45.00	4.00	15.00	10.00	15.00	5.00
ENFERMAGEM	FEMININO	NÃO BRANCA	SIM	HETEROSSEXUAL	SEM COMPANHEIRO	SÓ ESTUDA	NÃO MORA SÓ	3.00	67.00	0.00	15.00	14.00	18.00	8.00
ENFERMAGEM	FEMININO	NÃO BRANCA	SIM	HETEROSSEXUAL	COM COMPANHEIRO	ESTUDA E TRABALHA	NÃO MORA SÓ	3.00	62.00	0.00	14.00	12.00	15.00	10.00
ENFERMAGEM	FEMININO	BRANCA	NÃO	HETEROSSEXUAL	SEM COMPANHEIRO	SÓ ESTUDA	NÃO MORA SÓ	3.00	71.00	0.00	10.00	10.00	10.00	8.00
ENFERMAGEM	FEMININO	NÃO BRANCA	SIM	MINORIAS SEXUAIS	SEM COMPANHEIRO	SÓ ESTUDA	NÃO MORA SÓ	3.00	46.00	1.00	20.00	18.00	22.00	7.00
ENFERMAGEM	FEMININO	NÃO BRANCA	SIM	HETEROSSEXUAL	SEM COMPANHEIRO	SÓ ESTUDA	NÃO MORA SÓ	3.00	38.00	14.00	21.00	19.00	19.00	18.00
ENFERMAGEM	FEMININO	NÃO BRANCA	SIM	HETEROSSEXUAL	SEM COMPANHEIRO	SÓ ESTUDA	NÃO MORA SÓ	3.00	79.00	0.00	7.00	9.00	9.00	8.00
ENFERMAGEM	FEMININO	BRANCA	SIM	HETEROSSEXUAL	COM COMPANHEIRO	SÓ ESTUDA	NÃO MORA SÓ	3.00	39.00	0.00	18.00	13.00	23.00	11.00

ENFERMAGEM	FEMININO	NÃO BRANCA	SIM	HETEROSSEXUAL	COM COMPANHEIRO	SÓ ESTUDA	NÃO MORA SÓ	3.00	66.00	0.00	14.00	15.00	18.00	14.00
ENFERMAGEM	FEMININO	BRANCA	SIM	HETEROSSEXUAL	COM COMPANHEIRO	SÓ ESTUDA	NÃO MORA SÓ	3.00	69.00	0.00	9.00	8.00	16.00	11.00
MEDICINA	FEMININO	BRANCA	NÃO	HETEROSSEXUAL	SEM COMPANHEIRO	SÓ ESTUDA	MORA SÓ	0.00	39.00	0.00			19.00	13.00
MEDICINA	FEMININO	NÃO BRANCA	SIM	HETEROSSEXUAL	COM COMPANHEIRO	SÓ ESTUDA	NÃO MORA SÓ	0.00	32.00	0.00	15.00	10.00	22.00	10.00
MEDICINA	MASCULINO	NÃO BRANCA	SIM			ESTUDA E TRABALHA	NÃO MORA SÓ	0.00	58.00	0.00	9.00	7.00	17.00	11.00
MEDICINA	FEMININO	BRANCA	SIM	HETEROSSEXUAL	SEM COMPANHEIRO	SÓ ESTUDA	NÃO MORA SÓ	0.00	45.00	0.00	11.00	22.00	19.00	14.00
MEDICINA	MASCULINO	BRANCA	NÃO	HETEROSSEXUAL	SEM COMPANHEIRO	SÓ ESTUDA	MORA SÓ	0.00	53.00	0.00	12.00	11.00	17.00	12.00
MEDICINA	MASCULINO	BRANCA	NÃO	MINORIAS SEXUAIS	SEM COMPANHEIRO	SÓ ESTUDA	NÃO MORA SÓ	0.00	48.00	1.00	16.00	20.00	19.00	15.00
MEDICINA	FEMININO	BRANCA	SIM	HETEROSSEXUAL	COM COMPANHEIRO	SÓ ESTUDA	MORA SÓ	0.00	72.00	0.00	11.00	8.00	9.00	7.00
MEDICINA	FEMININO	BRANCA	SIM	HETEROSSEXUAL	SEM COMPANHEIRO	SÓ ESTUDA	NÃO MORA SÓ	0.00	56.00	1.00	20.00	14.00	17.00	9.00
MEDICINA	MASCULINO	BRANCA	NÃO	HETEROSSEXUAL	SEM COMPANHEIRO	SÓ ESTUDA	NÃO MORA SÓ	0.00	60.00	0.00	10.00	11.00	19.00	9.00
MEDICINA	MASCULINO	NÃO BRANCA	NÃO	MINORIAS SEXUAIS	COM COMPANHEIRO	SÓ ESTUDA	NÃO MORA SÓ	0.00	76.00	0.00	10.00	11.00	12.00	0.00
MEDICINA	MASCULINO	BRANCA	NÃO	HETEROSSEXUAL	SEM COMPANHEIRO	SÓ ESTUDA	NÃO MORA SÓ	0.00	54.00	0.00	11.00	10.00	17.00	7.00
MEDICINA	FEMININO	BRANCA	SIM	MINORIAS SEXUAIS	SEM COMPANHEIRO	SÓ ESTUDA	NÃO MORA SÓ	0.00	54.00	0.00	17.00	14.00	21.00	12.00
MEDICINA	FEMININO	BRANCA	NÃO	MINORIAS SEXUAIS	SEM COMPANHEIRO	SÓ ESTUDA	NÃO MORA SÓ	0.00	57.00	0.00	11.00	11.00	17.00	2.00
MEDICINA	FEMININO	BRANCA	SIM	HETEROSSEXUAL	SEM COMPANHEIRO	SÓ ESTUDA	MORA SÓ	0.00	54.00	0.00	12.00	18.00	18.00	11.00
MEDICINA	FEMININO	BRANCA	SIM	HETEROSSEXUAL	SEM COMPANHEIRO	SÓ ESTUDA	MORA SÓ	0.00	42.00	0.00	16.00	12.00	28.00	13.00
MEDICINA	FEMININO	BRANCA	NÃO	HETEROSSEXUAL	COM COMPANHEIRO	SÓ ESTUDA	NÃO MORA SÓ	0.00	38.00	0.00	10.00	9.00	16.00	10.00
MEDICINA	MASCULINO	BRANCA	SIM	HETEROSSEXUAL	SEM COMPANHEIRO	SÓ ESTUDA	MORA SÓ	0.00	57.00	0.00	9.00	9.00	11.00	11.00
MEDICINA	MASCULINO	NÃO BRANCA	SIM	HETEROSSEXUAL	SEM COMPANHEIRO	SÓ ESTUDA	NÃO MORA SÓ	0.00	52.00	0.00	8.00	8.00	13.00	9.00
MEDICINA	FEMININO	NÃO BRANCA	SIM	HETEROSSEXUAL	COM COMPANHEIRO	ESTUDA E TRABALHA	NÃO MORA SÓ	0.00	61.00	0.00	18.00	15.00	21.00	10.00

MEDICINA	FEMININO	NÃO BRANCA	SIM	MINORIAS SEXUAIS	SEM COMPANHEIRO	SÓ ESTUDA	NÃO MORA SÓ	0.00	48.00	2.00	12.00	11.00	20.00	12.00
MEDICINA	FEMININO	BRANCA	NÃO	MINORIAS SEXUAIS	SEM COMPANHEIRO	SÓ ESTUDA	NÃO MORA SÓ	0.00	54.00	0.00	9.00	9.00	11.00	9.00
MEDICINA	FEMININO	BRANCA	SIM	HETEROSSEXUAL	SEM COMPANHEIRO	SÓ ESTUDA	NÃO MORA SÓ	0.00	40.00	0.00	22.00	22.00	28.00	6.00
MEDICINA	FEMININO	BRANCA	NÃO	HETEROSSEXUAL	COM COMPANHEIRO	SÓ ESTUDA	MORA SÓ	0.00	38.00	0.00	18.00	11.00	19.00	4.00
MEDICINA	MASCULINO	NÃO BRANCA	NÃO	MINORIAS SEXUAIS	SEM COMPANHEIRO	SÓ ESTUDA	MORA SÓ	0.00	43.00	0.00	17.00	23.00	23.00	15.00
MEDICINA	FEMININO	BRANCA	SIM	HETEROSSEXUAL	SEM COMPANHEIRO	SÓ ESTUDA	NÃO MORA SÓ	0.00	44.00	0.00	14.00	13.00	26.00	6.00
MEDICINA	FEMININO	BRANCA	SIM	HETEROSSEXUAL	SEM COMPANHEIRO	SÓ ESTUDA	NÃO MORA SÓ	0.00	58.00	1.00	17.00	10.00	16.00	17.00
MEDICINA	FEMININO	BRANCA	NÃO	HETEROSSEXUAL	COM COMPANHEIRO	SÓ ESTUDA	NÃO MORA SÓ	0.00	26.00	33.00	23.00	12.00	23.00	16.00
MEDICINA	FEMININO	NÃO BRANCA	NÃO	MINORIAS SEXUAIS	SEM COMPANHEIRO	SÓ ESTUDA	NÃO MORA SÓ	0.00	36.00	7.00	24.00	22.00	24.00	14.00
MEDICINA	FEMININO	NÃO BRANCA	SIM	HETEROSSEXUAL	COM COMPANHEIRO	ESTUDA E TRABALHA	NÃO MORA SÓ	0.00	59.00	4.00	10.00	8.00	13.00	6.00
MEDICINA	MASCULINO	BRANCA	SIM	MINORIAS SEXUAIS	SEM COMPANHEIRO	SÓ ESTUDA	NÃO MORA SÓ	0.00	61.00	19.00	12.00	15.00	20.00	15.00
MEDICINA	FEMININO	NÃO BRANCA	SIM	HETEROSSEXUAL	SEM COMPANHEIRO	SÓ ESTUDA	NÃO MORA SÓ	0.00	35.00	1.00	22.00	24.00	24.00	13.00
MEDICINA	FEMININO	BRANCA	SIM	HETEROSSEXUAL	COM COMPANHEIRO	ESTUDA E TRABALHA	NÃO MORA SÓ	0.00	42.00	4.00	13.00	15.00	15.00	11.00
MEDICINA	FEMININO	NÃO BRANCA	SIM	HETEROSSEXUAL	SEM COMPANHEIRO	SÓ ESTUDA	MORA SÓ	0.00	36.00	3.00	16.00	15.00	19.00	17.00
MEDICINA	FEMININO	NÃO BRANCA	NÃO	MINORIAS SEXUAIS	SEM COMPANHEIRO	SÓ ESTUDA	NÃO MORA SÓ	0.00	31.00	17.00	17.00	11.00	13.00	2.00
MEDICINA	FEMININO	BRANCA	NÃO	HETEROSSEXUAL	SEM COMPANHEIRO	SÓ ESTUDA	MORA SÓ	0.00	28.00	0.00	12.00	10.00	17.00	7.00
MEDICINA	MASCULINO	BRANCA	SIM	HETEROSSEXUAL	SEM COMPANHEIRO	SÓ ESTUDA	MORA SÓ	0.00	42.00	0.00	15.00	15.00	17.00	17.00
MEDICINA	MASCULINO	BRANCA	SIM	HETEROSSEXUAL	SEM COMPANHEIRO	SÓ ESTUDA	NÃO MORA SÓ	0.00	55.00	0.00	14.00	14.00	22.00	10.00
MEDICINA	MASCULINO	BRANCA	NÃO	MINORIAS SEXUAIS	SEM COMPANHEIRO	SÓ ESTUDA	MORA SÓ	0.00	44.00	0.00	11.00	11.00	18.00	6.00
MEDICINA	FEMININO	BRANCA	SIM	HETEROSSEXUAL	SEM COMPANHEIRO	SÓ ESTUDA	NÃO MORA SÓ	0.00	42.00	0.00	15.00	16.00	23.00	11.00
MEDICINA	MASCULINO	NÃO BRANCA	SIM	HETEROSSEXUAL	SEM COMPANHEIRO	ESTUDA E TRABALHA	MORA SÓ	0.00	62.00	0.00	8.00	10.00	7.00	5.00

MEDICINA	MASCULINO	NÃO BRANCA	SIM	HETEROSSEXUAL	SEM COMPANHEIRO	SÓ ESTUDA	NÃO MORA SÓ	0.00	49.00	0.00	12.00	11.00	12.00	14.00
MEDICINA	MASCULINO	NÃO BRANCA	SIM	HETEROSSEXUAL	SEM COMPANHEIRO	SÓ ESTUDA	NÃO MORA SÓ	0.00	53.00	0.00	9.00	9.00	16.00	8.00
MEDICINA	FEMININO	BRANCA	SIM	HETEROSSEXUAL	COM COMPANHEIRO	SÓ ESTUDA	MORA SÓ	0.00	65.00	0.00	9.00	7.00	12.00	13.00
MEDICINA	FEMININO	NÃO BRANCA	SIM	HETEROSSEXUAL	SEM COMPANHEIRO	SÓ ESTUDA	NÃO MORA SÓ	0.00	50.00	0.00	12.00	20.00	20.00	8.00
MEDICINA	MASCULINO	BRANCA	SIM	HETEROSSEXUAL	SEM COMPANHEIRO	SÓ ESTUDA	MORA SÓ	0.00	52.00	0.00	14.00	19.00	20.00	4.00
MEDICINA	FEMININO	BRANCA	SIM	HETEROSSEXUAL	SEM COMPANHEIRO	SÓ ESTUDA	NÃO MORA SÓ	1.00	47.00	0.00	12.00	8.00	18.00	9.00
MEDICINA	FEMININO	NÃO BRANCA	SIM	HETEROSSEXUAL	SEM COMPANHEIRO	SÓ ESTUDA	NÃO MORA SÓ	1.00	40.00	0.00	14.00	14.00	20.00	15.00
MEDICINA	FEMININO	NÃO BRANCA	SIM	HETEROSSEXUAL	COM COMPANHEIRO	SÓ ESTUDA	NÃO MORA SÓ	1.00	82.00	0.00	8.00	9.00	11.00	14.00
MEDICINA	FEMININO	NÃO BRANCA	NÃO	HETEROSSEXUAL	SEM COMPANHEIRO	ESTUDA E TRABALHA	NÃO MORA SÓ	1.00	46.00	0.00	14.00	10.00	14.00	8.00
MEDICINA	MASCULINO	NÃO BRANCA	SIM	HETEROSSEXUAL	SEM COMPANHEIRO	SÓ ESTUDA	NÃO MORA SÓ	1.00	87.00	0.00	7.00	8.00	7.00	9.00
MEDICINA	MASCULINO	NÃO BRANCA	SIM	HETEROSSEXUAL	SEM COMPANHEIRO	SÓ ESTUDA	NÃO MORA SÓ	1.00	70.00	0.00	11.00	13.00	12.00	7.00
MEDICINA	FEMININO	NÃO BRANCA	SIM	HETEROSSEXUAL	SEM COMPANHEIRO	SÓ ESTUDA	NÃO MORA SÓ	1.00	52.00	0.00	12.00	11.00	19.00	9.00
MEDICINA	MASCULINO	BRANCA	NÃO	HETEROSSEXUAL	SEM COMPANHEIRO	SÓ ESTUDA	MORA SÓ	1.00	61.00	0.00	9.00	7.00	7.00	13.00
MEDICINA	FEMININO	NÃO BRANCA	NÃO	MINORIAS SEXUAIS	SEM COMPANHEIRO	SÓ ESTUDA	MORA SÓ	1.00	43.00	16.00	22.00	15.00	24.00	13.00
MEDICINA	FEMININO	BRANCA	NÃO	MINORIAS SEXUAIS	SEM COMPANHEIRO	SÓ ESTUDA	MORA SÓ	1.00	61.00	0.00	9.00	10.00	15.00	8.00
MEDICINA	MASCULINO	NÃO BRANCA	NÃO	MINORIAS SEXUAIS	SEM COMPANHEIRO	SÓ ESTUDA	NÃO MORA SÓ	1.00	52.00	3.00	21.00	11.00	20.00	16.00
MEDICINA	MASCULINO	BRANCA	NÃO	HETEROSSEXUAL	SEM COMPANHEIRO	SÓ ESTUDA	NÃO MORA SÓ	1.00	44.00	0.00	17.00	17.00	17.00	
MEDICINA	FEMININO	NÃO BRANCA	SIM	HETEROSSEXUAL	SEM COMPANHEIRO	SÓ ESTUDA	NÃO MORA SÓ	1.00	47.00	0.00	15.00	13.00	18.00	16.00
MEDICINA	MASCULINO	BRANCA	SIM	HETEROSSEXUAL	SEM COMPANHEIRO	SÓ ESTUDA	MORA SÓ	1.00	58.00	7.00	15.00	9.00	13.00	7.00
MEDICINA	MASCULINO	BRANCA	SIM	MINORIAS SEXUAIS	SEM COMPANHEIRO	SÓ ESTUDA	NÃO MORA SÓ	1.00	56.00	19.00	11.00	13.00	14.00	5.00
MEDICINA	MASCULINO	BRANCA	SIM	HETEROSSEXUAL	COM COMPANHEIRO	SÓ ESTUDA	NÃO MORA SÓ	5.00	28.00	0.00	13.00	8.00	14.00	7.00

MEDICINA	MASCULINO	BRANCA	NÃO	HETEROSSEXUAL	SEM COMPANHEIRO	SÓ ESTUDA	NÃO MORA SÓ	5.00	46.00	17.00	22.00	9.00	16.00	5.00
MEDICINA	FEMININO	BRANCA	SIM	HETEROSSEXUAL	COM COMPANHEIRO	SÓ ESTUDA	NÃO MORA SÓ	5.00	29.00	0.00	16.00	13.00	25.00	20.00
MEDICINA	FEMININO	NÃO BRANCA	NÃO	HETEROSSEXUAL	SEM COMPANHEIRO	SÓ ESTUDA	NÃO MORA SÓ	5.00	38.00	23.00	20.00	19.00	24.00	11.00
MEDICINA	MASCULINO	NÃO BRANCA	NÃO	HETEROSSEXUAL	SEM COMPANHEIRO	SÓ ESTUDA	NÃO MORA SÓ	5.00	63.00	0.00	7.00	7.00	15.00	9.00
MEDICINA	FEMININO	BRANCA	SIM	HETEROSSEXUAL	SEM COMPANHEIRO	SÓ ESTUDA	NÃO MORA SÓ	5.00	59.00	0.00	10.00	10.00	13.00	10.00
MEDICINA	MASCULINO	BRANCA	NÃO	HETEROSSEXUAL	SEM COMPANHEIRO	SÓ ESTUDA	NÃO MORA SÓ	5.00	60.00	0.00	10.00	9.00	15.00	11.00
MEDICINA	FEMININO	BRANCA	NÃO	HETEROSSEXUAL	COM COMPANHEIRO	SÓ ESTUDA	NÃO MORA SÓ	5.00	35.00	0.00	13.00	10.00	21.00	7.00
MEDICINA	MASCULINO	NÃO BRANCA	SIM	HETEROSSEXUAL	COM COMPANHEIRO	SÓ ESTUDA	NÃO MORA SÓ	5.00	69.00	0.00	9.00	8.00	11.00	7.00
MEDICINA	MASCULINO	NÃO BRANCA	SIM	HETEROSSEXUAL	COM COMPANHEIRO	ESTUDA E TRABALHA	NÃO MORA SÓ	5.00	45.00	0.00	19.00	8.00	13.00	8.00
MEDICINA	FEMININO	BRANCA	SIM	HETEROSSEXUAL	SEM COMPANHEIRO	SÓ ESTUDA	MORA SÓ	5.00	54.00	0.00	9.00	7.00	15.00	8.00
MEDICINA	FEMININO	BRANCA	SIM	HETEROSSEXUAL	SEM COMPANHEIRO	SÓ ESTUDA	NÃO MORA SÓ	5.00	61.00	4.00	10.00	11.00	18.00	7.00
MEDICINA	FEMININO	BRANCA	SIM	HETEROSSEXUAL	SEM COMPANHEIRO	SÓ ESTUDA	NÃO MORA SÓ	5.00	52.00	0.00	13.00	8.00	18.00	14.00
MEDICINA	MASCULINO	NÃO BRANCA	SIM	HETEROSSEXUAL	COM COMPANHEIRO	SÓ ESTUDA	NÃO MORA SÓ	5.00	42.00	0.00	16.00	13.00	25.00	11.00
MEDICINA	MASCULINO	BRANCA	SIM	MINORIAS SEXUAIS	COM COMPANHEIRO	SÓ ESTUDA	NÃO MORA SÓ	5.00	72.00	0.00	11.00	10.00	21.00	6.00
MEDICINA	FEMININO	BRANCA	NÃO	MINORIAS SEXUAIS	SEM COMPANHEIRO	ESTUDA E TRABALHA	NÃO MORA SÓ	5.00	48.00	0.00	16.00	12.00	19.00	17.00
MEDICINA	MASCULINO	NÃO BRANCA	NÃO	HETEROSSEXUAL	COM COMPANHEIRO	SÓ ESTUDA	NÃO MORA SÓ	5.00	44.00	0.00	10.00	8.00	10.00	8.00
MEDICINA	FEMININO	NÃO BRANCA	NÃO	HETEROSSEXUAL	SEM COMPANHEIRO	SÓ ESTUDA	NÃO MORA SÓ	5.00	51.00	6.00	9.00	11.00	18.00	10.00
MEDICINA	MASCULINO	NÃO BRANCA	SIM	HETEROSSEXUAL	COM COMPANHEIRO	SÓ ESTUDA		5.00	48.00	0.00	10.00	20.00	22.00	8.00
MEDICINA	MASCULINO	NÃO BRANCA	SIM	HETEROSSEXUAL	SEM COMPANHEIRO	ESTUDA E TRABALHA	MORA SÓ	5.00	76.00	0.00	8.00	7.00	9.00	6.00
MEDICINA	FEMININO	BRANCA	NÃO	MINORIAS SEXUAIS	SEM COMPANHEIRO	SÓ ESTUDA	MORA SÓ	5.00	52.00	0.00	19.00	11.00	16.00	10.00
MEDICINA	FEMININO	NÃO BRANCA	NÃO	HETEROSSEXUAL	COM COMPANHEIRO	SÓ ESTUDA	NÃO MORA SÓ	5.00	48.00	0.00	14.00	9.00	18.00	13.00

MEDICINA	MASCULINO	BRANCA	NÃO	MINORIAS SEXUAIS	COM COMPANHEIRO	SÓ ESTUDA	NÃO MORA SÓ	5.00	41.00	2.00	17.00	11.00	16.00	4.00
MEDICINA	FEMININO	NÃO BRANCA	NÃO	HETEROSSEXUAL	SEM COMPANHEIRO	SÓ ESTUDA	NÃO MORA SÓ	5.00	47.00	3.00	22.00	17.00	27.00	13.00
MEDICINA	MASCULINO	NÃO BRANCA	NÃO	HETEROSSEXUAL	COM COMPANHEIRO	ESTUDA E TRABALHA	MORA SÓ	5.00	49.00	0.00	15.00	9.00	14.00	7.00
MEDICINA	MASCULINO	NÃO BRANCA	SIM	HETEROSSEXUAL	SEM COMPANHEIRO	ESTUDA E TRABALHA	MORA SÓ	5.00	67.00	0.00	10.00	9.00	10.00	7.00
MEDICINA	MASCULINO	BRANCA	SIM	HETEROSSEXUAL	SEM COMPANHEIRO	SÓ ESTUDA	NÃO MORA SÓ	5.00	44.00	0.00	23.00	10.00	18.00	8.00
MEDICINA	FEMININO	BRANCA	2.00	MINORIAS SEXUAIS	SEM COMPANHEIRO	SÓ ESTUDA	NÃO MORA SÓ	5.00	54.00	0.00	19.00	15.00	18.00	9.00
MEDICINA	MASCULINO	BRANCA	NÃO	HETEROSSEXUAL	SEM COMPANHEIRO	SÓ ESTUDA	NÃO MORA SÓ	5.00	61.00	0.00	12.00	16.00	18.00	11.00
MEDICINA	FEMININO	BRANCA	NÃO	MINORIAS SEXUAIS	COM COMPANHEIRO	ESTUDA E TRABALHA	NÃO MORA SÓ	5.00	40.00	5.00	11.00	11.00	15.00	10.00
MEDICINA	FEMININO	NÃO BRANCA	SIM	MINORIAS SEXUAIS	COM COMPANHEIRO	ESTUDA E TRABALHA	NÃO MORA SÓ	5.00	64.00				17.00	17.00
MEDICINA	MASCULINO	BRANCA	NÃO	HETEROSSEXUAL	COM COMPANHEIRO	SÓ ESTUDA	NÃO MORA SÓ	5.00	57.00	0.00	7.00	7.00	10.00	9.00
MEDICINA	FEMININO	BRANCA	SIM	HETEROSSEXUAL	COM COMPANHEIRO	SÓ ESTUDA	MORA SÓ	5.00	70.00	0.00	15.00	8.00	14.00	6.00
MEDICINA	FEMININO	BRANCA	SIM	HETEROSSEXUAL	SEM COMPANHEIRO	ESTUDA E TRABALHA	NÃO MORA SÓ	5.00	63.00	0.00	9.00	10.00	14.00	14.00
MEDICINA	MASCULINO	BRANCA	NÃO	MINORIAS SEXUAIS	SEM COMPANHEIRO	ESTUDA E TRABALHA	MORA SÓ	5.00	40.00	13.00	10.00	13.00	18.00	15.00
MEDICINA	FEMININO	BRANCA	NÃO	MINORIAS SEXUAIS	SEM COMPANHEIRO	ESTUDA E TRABALHA	NÃO MORA SÓ	5.00	56.00	0.00	9.00	8.00	9.00	13.00
MEDICINA	MASCULINO	NÃO BRANCA	SIM	HETEROSSEXUAL	SEM COMPANHEIRO	ESTUDA E TRABALHA	MORA SÓ	5.00	51.00	9.00	16.00	7.00	15.00	12.00
MEDICINA	FEMININO	NÃO BRANCA	SIM	MINORIAS SEXUAIS	COM COMPANHEIRO	SÓ ESTUDA	NÃO MORA SÓ	5.00	29.00	2.00	25.00	22.00	20.00	18.00
MEDICINA	MASCULINO	NÃO BRANCA	SIM	HETEROSSEXUAL	COM COMPANHEIRO	SÓ ESTUDA	MORA SÓ	5.00	56.00	0.00	7.00	9.00	10.00	9.00
MEDICINA	FEMININO	BRANCA	SIM	HETEROSSEXUAL	SEM COMPANHEIRO	SÓ ESTUDA	MORA SÓ	5.00	71.00	0.00	8.00	11.00	15.00	6.00
MEDICINA	FEMININO	BRANCA	SIM	HETEROSSEXUAL	COM COMPANHEIRO	SÓ ESTUDA	NÃO MORA SÓ	5.00	58.00	0.00	16.00	13.00	17.00	5.00
MEDICINA	MASCULINO	NÃO BRANCA	NÃO	HETEROSSEXUAL	SEM COMPANHEIRO	SÓ ESTUDA	MORA SÓ	5.00	65.00	0.00	20.00	7.00	17.00	16.00
MEDICINA	FEMININO	NÃO BRANCA	SIM	HETEROSSEXUAL	COM COMPANHEIRO	SÓ ESTUDA	NÃO MORA SÓ	3.00	36.00	0.00	15.00	16.00	21.00	4.00

MEDICINA	MASCULINO	NÃO BRANCA	NÃO	HETEROSSEXUAL	SEM COMPANHEIRO	SÓ ESTUDA	NÃO MORA SÓ	3.00	48.00	1.00	25.00	14.00	17.00	8.00
MEDICINA	FEMININO	BRANCA	SIM	HETEROSSEXUAL	COM COMPANHEIRO	SÓ ESTUDA	NÃO MORA SÓ	3.00	48.00	0.00	8.00	7.00	16.00	17.00
MEDICINA	MASCULINO	NÃO BRANCA	SIM	HETEROSSEXUAL	SEM COMPANHEIRO	SÓ ESTUDA	MORA SÓ	3.00	64.00	0.00	8.00	7.00	11.00	7.00
MEDICINA	MASCULINO	NÃO BRANCA	NÃO	MINORIAS SEXUAIS	COM COMPANHEIRO	SÓ ESTUDA	NÃO MORA SÓ	3.00	37.00	0.00	18.00	10.00	17.00	11.00
MEDICINA	MASCULINO	BRANCA	SIM	MINORIAS SEXUAIS	COM COMPANHEIRO	SÓ ESTUDA	NÃO MORA SÓ	3.00	43.00	0.00	15.00	19.00	23.00	14.00
MEDICINA	MASCULINO	BRANCA	SIM	HETEROSSEXUAL	SEM COMPANHEIRO	SÓ ESTUDA	NÃO MORA SÓ	3.00	60.00	0.00	7.00	8.00	11.00	7.00
MEDICINA	FEMININO	BRANCA	NÃO	HETEROSSEXUAL	SEM COMPANHEIRO	SÓ ESTUDA	MORA SÓ	3.00	43.00	0.00	16.00	8.00	22.00	11.00
MEDICINA	MASCULINO	NÃO BRANCA	SIM	HETEROSSEXUAL	SEM COMPANHEIRO	ESTUDA E TRABALHA	MORA SÓ	3.00	47.00	0.00	9.00	11.00	19.00	9.00
MEDICINA	FEMININO	BRANCA	SIM	HETEROSSEXUAL	SEM COMPANHEIRO	SÓ ESTUDA	NÃO MORA SÓ	3.00	57.00	0.00	10.00	10.00	12.00	7.00
MEDICINA	MASCULINO	BRANCA	NÃO	HETEROSSEXUAL	COM COMPANHEIRO	SÓ ESTUDA	NÃO MORA SÓ	3.00	36.00	0.00	16.00	9.00	24.00	8.00
MEDICINA	MASCULINO	BRANCA	SIM	HETEROSSEXUAL	SEM COMPANHEIRO	SÓ ESTUDA	MORA SÓ	3.00	62.00	0.00	8.00	9.00	10.00	7.00
MEDICINA	FEMININO	NÃO BRANCA	SIM	HETEROSSEXUAL	SEM COMPANHEIRO	SÓ ESTUDA	NÃO MORA SÓ	3.00	55.00	0.00	9.00	7.00	14.00	12.00
MEDICINA	MASCULINO	BRANCA	NÃO	MINORIAS SEXUAIS	SEM COMPANHEIRO		NÃO MORA SÓ	3.00	51.00	9.00	22.00	10.00	25.00	10.00
MEDICINA	FEMININO	BRANCA	SIM	HETEROSSEXUAL	COM COMPANHEIRO	SÓ ESTUDA	MORA SÓ	3.00	61.00	0.00	8.00	8.00	11.00	6.00
MEDICINA	MASCULINO	BRANCA	NÃO	MINORIAS SEXUAIS	SEM COMPANHEIRO	SÓ ESTUDA	MORA SÓ	3.00	57.00	5.00	18.00	9.00	13.00	12.00
MEDICINA	MASCULINO	BRANCA	SIM	HETEROSSEXUAL	SEM COMPANHEIRO	ESTUDA E TRABALHA	NÃO MORA SÓ	3.00	50.00	2.00	15.00	15.00	21.00	12.00
MEDICINA	FEMININO	NÃO BRANCA	SIM	HETEROSSEXUAL	SEM COMPANHEIRO	SÓ ESTUDA	MORA SÓ	3.00	38.00	8.00	14.00	16.00	24.00	8.00
ENFERMAGEM	FEMININO	NÃO BRANCA	SIM	HETEROSSEXUAL	SEM COMPANHEIRO	SÓ ESTUDA	MORA SÓ	5.00	75.00	0.00	14.00	14.00	15.00	10.00
ENFERMAGEM	FEMININO	NÃO BRANCA	SIM	MINORIAS SEXUAIS	SEM COMPANHEIRO	SÓ ESTUDA	NÃO MORA SÓ	5.00	66.00	0.00	15.00	18.00	18.00	6.00
ENFERMAGEM	FEMININO	BRANCA	NÃO	HETEROSSEXUAL	COM COMPANHEIRO	SÓ ESTUDA	NÃO MORA SÓ	5.00	33.00	0.00	16.00	23.00	27.00	10.00
ENFERMAGEM	FEMININO	NÃO BRANCA	SIM	HETEROSSEXUAL	SEM COMPANHEIRO	SÓ ESTUDA	NÃO MORA SÓ	5.00	31.00	1.00	18.00	20.00	23.00	21.00

ENFERMAGEM	FEMININO	NÃO BRANCA	SIM	HETEROSSEXUAL	SEM COMPANHEIRO	SÓ ESTUDA	NÃO MORA SÓ	5.00	55.00	17.00	20.00	16.00	22.00	6.00
ENFERMAGEM	FEMININO	NÃO BRANCA	NÃO	HETEROSSEXUAL	SEM COMPANHEIRO	SÓ ESTUDA	NÃO MORA SÓ	5.00	42.00	7.00	15.00	10.00	14.00	11.00
ENFERMAGEM	FEMININO	NÃO BRANCA	SIM	HETEROSSEXUAL	COM COMPANHEIRO		NÃO MORA SÓ	5.00	47.00	17.00	19.00	14.00	18.00	14.00
