# Supplementary material for: Indicators of emotional distress and mindfulness in undergraduate students: a cross-sectional study
Source: Rev Bras Enferm. 2024 Oct 7;77(5):e20230499. doi: 10.1590/0034-7167-2023-0499 (PMC11458143; doi:10.1590/0034-7167-2023-0499)
Supplement: Translation

# Translation: Indicadores de sofrimento emocional e atenção plena em estudantes universitários: estudo transversal

**DOI:** 10.1590/0034-7167-2023-0499pt

**Authors:** Vanessa Ferraz Leite, Moisés Kogien, Margani Cadore Weis Maia, Marina Nolli Bittencourt, Larissa de Almeida Rézio, Samira Reschetti Marcon

**Affiliations:** I Universidade Federal de Mato Grosso. Cuiabá, Mato Grosso, Brasil

**Keywords:** Estudantes, Universidades, Saúde Mental, Sofrimento Emocional, Atenção Plena

## Abstract

**Objetivos:**

avaliar a associação entre indicadores de sofrimento emocional e atenção plena disposicional em estudantes da área da saúde de uma universidade pública federal brasileira.

**Métodos:**

estudo transversal, desenvolvido com universitários da área da saúde de uma instituição pública no período de maio a junho de 2022. Na análise, utilizou-se a regressão linear múltipla por meio do *software* SPSS.

**Resultados:**

a amostra mostrou-se majoritariamente feminina, ≤ 22 anos, não branca, cursando os primeiros semestres, com maior prevalência para o curso de medicina. Os estudantes apresentaram escores moderados de atenção plena disposicional. Observou-se que as variáveis estresse, depressão e risco de suicídio atual se associaram com a capacidade de atenção plena.

**Conclusões:**

conhecer os indicadores de sofrimento emocional que se relacionam ao potencial da atenção plena pode contribuir como um diagnóstico situacional para melhor delineamento de estratégias que promovam a melhora dos indicadores emocionais de estudantes da área da saúde.

## INTRODUÇÃO

No campo da saúde, o termo “atenção plena” (*mindfulness*) *se* refere ao estado mental de atenção de forma consciente e deliberada à experiência do aqui e agora, com curiosidade, monitoramento, abertura, aceitação e sem julgamentos^(1, 2)^. Dessa forma, os estudos científicos compreendem a capacidade de atenção plena disposicional (APD) como um construto com características centrais de manutenção da capacidade de estar atento e consciente do momento presente na vida cotidiana^(3)^, e a sua relevância está na manutenção de capacidades que interagem com diferentes aspectos cognitivos, emocionais e motivacionais para o desenvolvimento pessoal e social^(4, 5)^. De natureza prática ou vivencial, a APD auxilia o indivíduo com o conhecimento sobre seus próprios processos de pensamento, ou seja, com a metacognição. Sabe-se que a metacognição, a atenção plena e a consciência sem julgamento do presente estão relacionadas, logo, ao cultivo da atenção plena, levando a uma maior consciência e compreensão das próprias experiências, pensamentos, emoções e sensações corporais^(1, 2)^.

No âmbito universitário, as diferentes atividades cognitivas às quais os estudantes estão expostos exigem concentração e a efetividade da atenção plena para facilitar a aprendizagem e ter bom rendimento acadêmico^(6)^. Contudo, determinados fatores, em associação, podem prejudicar o equilíbrio da autorregulação atencional e emocional dos estudantes^(7, 8)^. No que diz respeito aos estudantes da área da saúde, demandas como exigência de excelência acadêmica, necessidade de aquisição de competências clínicas para o cuidado^(9)^, o desenvolvimento de habilidades interpessoais para lidar com o sofrimento do outro e a competência para a solução de problemas se vinculam diretamente à habilidade atencional e regulação emocional^(10)^. Nesse contexto, a atenção plena, que se refere à capacidade de se manter atento intencional e mentalmente presente sem divagação^(2)^, torna-se um importante aliado para os estudantes na medida em que possibilita o processo de assimilação e enfrentamento emocional em meio à transitoriedade dos eventos^(11)^.

Cientificamente, a APD se correlaciona com diversos indicadores de saúde física e mental, como manutenção do equilíbrio do sistema nervoso autônomo simpático e parassimpático, índices elevados de afeto positivo, contentamento com a vida, vitalidade, afetos negativos e sintomatologias de quadros de sofrimento mental^(1)^. Embora o cultivo da APD seja um importante elemento no ambiente universitário à medida que propicia o aprendizado, alguns indicadores de sofrimento emocional (ISE) podem interferir na forma que os estudantes utilizam essa atenção plena, comprometendo, dessa forma, a sua efetividade.

ISE se referem a um conjunto de variáveis que permitem identificar as reações negativas contínuas que impedem o florescimento das potencialidades humanas^(12, 13)^. Entre eles, há as sintomatologias de depressão, ansiedade, estresse, ideias suicidas e sonolência diurna, e estudos científicos evidenciam uma elevação desses indicadores emocionais em estudantes universitários^(14, 15)^.

O aumento de alguns desses ISE e suas associações com a atenção plena afetam significativamente as atividades acadêmicas, uma vez que há um declínio da capacidade de APD^(11, 16)^. Dessa forma, apesar de o indivíduo apresentar considerável nível de atenção, em decorrência das inúmeras demandas ou circunstâncias, nem sempre bem assimiladas, acaba por desenvolver sintomas de depressão e ansiedade, o que pode resultar, por exemplo, em dificuldades acadêmicas. Contudo, a APD pode ser aperfeiçoada e cultivada por meio de programas de treinamento e intervenções^(2)^. Estudo com estudantes de medicina demonstrou que o estímulo da APD contribuiu na diminuição do esgotamento e promoveu bem-estar, podendo, portanto, ser incorporada a partir de treinamento e inserção em programas de graduação em saúde^(17)^, melhorando a capacidade de APD dos estudantes e, por conseguinte, seus potenciais efeitos salutares à saúde física e mental.

Considerando tal panorama, este estudo tem por hipótese que ISE são preditores negativos para a capacidade de APD de estudantes universitários da área da saúde, ou seja, quanto maior o nível de sofrimento emocional desses, menor será a capacidade de APD mensurada.

## OBJETIVOS

Avaliar a associação entre ISE e APD em estudantes da área da saúde de uma universidade pública federal brasileira.

## MÉTODOS

### Aspectos éticos

A pesquisa foi aprovada pelo Comitê de Ética em Pesquisa em Saúde e possui Certificado de Apresentação de Apreciação Ética.

### Desenho, período e local do estudo

Trata-se de estudo transversal norteado pela ferramenta *STrengthening the Reporting of OBservational studies in Epidemiology* (STROBE)^(18)^.

O estudo foi realizado no período de maio a junho de 2022 em uma universidade pública federal do centro-oeste brasileiro.

### População e amostra; critérios de inclusão e exclusão

A população-alvo foi composta de 833 estudantes dos cursos da área da saúde (enfermagem, medicina, nutrição e psicologia), regularmente matriculados no *campus* principal do local de estudo. Todos os estudantes que estavam em sala de aula nos dias de coleta foram convidados para participar do estudo, considerando-se o critério de inclusão idade igual ou superior a 18 anos completos. Não fizeram parte do estudo os acadêmicos em estágio ou internato, por não se encontrarem no *campus* no período da coleta. A amostra foi selecionada por conveniência, por meio da disponibilidade e aceite do estudante em participar do estudo. Foram recrutados 617 estudantes, sendo seis excluídos por incompletude dos questionários. Assim sendo, a amostra final foi constituída de 611 participantes (73,35% da população-alvo).

### Protocolo do estudo

Os dados foram coletados por meio de um instrumento fechado, de autopreenchimento, composto de variáveis socio-demográficas (sexo, idade, cor da pele autodeclarada, prática religiosa, orientação sexual, situação conjugal, de trabalho e moradia) e acadêmicas (semestre escolar e curso matriculado). O instrumento foi elaborado com base nos estudos do Fórum Nacional de Pró-reitores de Assuntos Comunitários e Estudantis^(19)^.

Utilizou-se a *Mindful Attention Awareness Scale* (MAAS)^(20)^, validada no Brasil^(3)^, para avaliar o nível de APD. É composta por 15 sentenças sobre o nível de atenção plena diante de situações cotidianas, com respostas em escala Likert de seis pontos, variando de 1 - quase sempre a 6 - quase nunca e escore total de 15 e 90 pontos. Neste estudo, a consistência interna foi de 0,86 (ω de McDonald).

Para avaliação dos sintomas de ansiedade, depressão e estresse, aplicou-se a *Depression, Anxiety, and Stress Scale* (DASS-21)^(21)^, validada no Brasil^(22)^, para uso em população universitária^(23)^. Possui 21 itens com respostas em escala tipo Likert de quatro pontos, divididos em três subescalas, cada uma com sete itens. Quanto maior o escore total de cada subescala, maiores são os sintomas de ansiedade, depressão e estresse. Neste estudo, a consistência interna para as subescalas de depressão, ansiedade e estresse foi de, respectivamente, 0,94, 0,89 e 0,99 (ω de McDonald).

Na coleta, utilizou-se, ainda, o módulo C da *Mini International Neuropsychiatry Interview* (MINI) para avaliação do risco de suicídio no último mês. Possui seis itens com respostas dicotômicas (sim ou não). Fornece um escore geral de 0 a 33 pontos. Neste estudo, os participantes foram estratificados do seguinte modo: escore 0 (sem risco de suicídio); escores entre 1 e 33 pontos (algum risco de suicídio atual). Trata-se de instrumento traduzido e validado para o contexto brasileiro com bom desempenho psicométrico^(24)^.

Por fim, avaliou-se o grau de sonolência diurna por meio da *Epworth Sleepiness Scale,* instrumento traduzido e validado para o contexto brasileiro^(25)^. Possui oito itens com respostas em escala tipo Likert de três pontos (0 - nenhuma probabilidade de pegar no sono a 3 - forte probabilidade de pegar no sono). Neste estudo, a consistência interna foi de 0,99 (ω de McDonald).

Anteriormente à aplicação dos instrumentos na coleta de dados, realizou-se um teste piloto, no mês de abril de 2022, com 56 alunos dos três primeiros semestres de um curso de ciências exatas da mesma instituição participante. No teste piloto, demonstrou-se boa aplicabilidade dos instrumentos, bem como bom tempo médio para o preenchimento (45 minutos).

A coleta de dados ocorreu presencialmente com visitas em sala de aula em dias definidos em cronograma prévio. Após autorização do professor em sala, realizou-se breve explicação da pesquisa e assinatura do Termo de Consentimento Livre Esclarecido (TCLE). O TCLE foi obtido de todos os indivíduos envolvidos no estudo por meio escrito. Os participantes responderam aos instrumentos de autopreenchimento de forma individual e, ao término, os depositaram em uma urna lacrada disposta em frente da sala, visando garantir a segurança do anonimato das respostas. Os dados foram coletados pela pesquisadora principal e oito acadêmicos dos cursos de enfermagem, medicina, psicologia e saúde coletiva devidamente treinados.

### Análise dos dados e estatística

Na análise, as variáveis contínuas foram expressas por meio de média e desvio padrão, enquanto que as variáveis categóricas foram apresentadas por frequências absolutas e relativas. Para realizar análises comparativas entre os escores médios de APD e os ISE, utilizaram-se testes t para grupos independentes, com magnitude de efeito estimada por meio do teste d de Cohen. Os Intervalos de Confiança de 95% foram obtidos por meio da técnica de *bootstrapping* com 1.000 reamostragens, que fornece Intervalos de Confiança corrigidos e acelerados por viés (*bias-corrected and accelerated confidence interval* (BCaCI)). Essa técnica foi utilizada para corrigir desvios da normalidade da distribuição amostral e diferenças entre os tamanhos dos grupos comparados.

Além disso, realizou-se análise múltipla de fatores associados por meio da técnica de regressão linear múltipla. Para a construção do modelo final adotado, todas as variáveis explicativas relacionadas ao sofrimento emocional foram incluídas no modelo e ajustadas para as características sociodemográficas e acadêmicas utilizadas neste estudo. Antes de assumir o modelo final, realizaram-se verificações dos pressupostos para a regressão linear múltipla, incluindo a conferência da normalidade da distribuição dos resíduos, a verificação da ausência de multicolinearidade por meio do *Variance Inflation Factor* (VIF) menor que dez e a confirmação da não ocorrência de autocorrelação de resíduos (Durbin-Watson =1,882). Todas as análises foram realizadas no *software Statistical Package for the Social Sciences* (SPSS), versão 27.

## RESULTADOS

A amostra de estudantes caracterizou-se como predominantemente feminina (73,3%), com mediana de idade ≤ 22 anos. A maior proporção estudantes declarou-se com cor da pele não branca (57,1%), orientação heterossexual (71,5%), sem companheiro(a) conjugal (67,4%) e não morando sozinho(a) durante sua formação acadêmica (81,5%). Além disso, a maior parte dos estudantes reportou realizar alguma prática religiosa (58,6%) e apenas dedicar-se aos estudos, sem necessidade de conciliar atividade laboral (77,9%) (Tabela 1).

**Tabela 1:** Perfil sociodemográfico e acadêmico dos estudantes da área da saúde de uma universidade pública da Região Centro-Oeste do Brasil (N = 611), Cuiabá, Mato Grosso, Brasil, 2022

Características sociodemográficas/acadêmicas	n (%)
Sexo	
Feminino	448 (73,3%)
Masculino	161 (26,4%)
Não informaram	02 (0,3%)
Idade mediana	
≤ 22 anos	384 (62,8%)
> 23 anos	227 (36,3%)
Cor da pele autodeclarada	
Não branca	349 (57,1%)
Branca	262 (42,9%)
Orientação sexual	
Heterossexual	437 (71,5%)
Orientações sexuais minoritárias	168 (27,5%)
Não informaram	06 (1,0%)
Prática religiosa	
Sim	358 (58,6%)
Não	252 (41,2%)
Não informaram	01 (0,2%)
*Status* conjugal	
Com companheiro(a)	194 (31,8%)
Sem companheiro(a)	412 (67,4%)
Não informaram	05 (0,8%)
Situação de moradia	
Mora sozinho(a)	110 (18,0%)
Não mora sozinho(a)	498 (81,5%)
Não informaram	03 (0,5%)
Situação laboral	
Trabalha e estuda	128 (21,0%)
Somente estuda	476 (77,9%)
Não informaram	07 (1,1%)
Período de curso	
1° ou 2° semestre	163 (26,7%)
3° ou 4° semestre	194 (31,8%)
5° ou 6° semestre	143 (23,4%)
7° ou 8° semestre	107 (17,5%)
Não informaram	04 (0,7%)
Curso	
Enfermagem	136 (22,3%)
Nutrição	114 (18,7%)
Psicologia	176 (28,8%)
Medicina	185 (30,3%)

Em relação ao perfil acadêmico, participaram estudantes do 1° ao 8° semestre, com maior participação de estudantes do 3° ou 4° semestres (31,8%). Estudantes do curso de medicina predominaram na amostra final analisada (30,3%).

A amostra total apresentou níveis moderados de APD (**x̅** = 51,75; desvio padrão = 12,67). Análise por subgrupos criados a partir dos ISE demonstraram diferenças entre os estudantes com evidências de sofrimento, quando comparados com aqueles sem (Tabela 2).

**Tabela 2:** Relação entre indicadores de sofrimento emocional e atenção plena disposicional em estudantes da área da saúde, Cuiabá, Mato Grosso, Brasil, 2022

Indicadores de sofrimento emocional	Frequências n (%)	Média (desvio padrão)	Atenção plena disposicional		d de Cohen
			t	Valor de *p* (BCa IC95%)	
Depressão					
Sem sintomas	287 (47,0%)	57,08 (12,00)	10,872	<0,001 (8,61; 12,04)	0,88
Com sintomas	319 (52,2%)	46,84 (11,18)			
Não responderam**	05 (0,8%)				
Ansiedade					
Sem sintomas	158 (25,9%)	56,95 (12,41)	6,273	<0,001 (4,89; 9,70)	0,58
Com sintomas	448 (73,3%)	49,83 (12,21)			
Não responderam**	05 (0,8%)				
Estresse					
Sem sintomas	373 (61,0%)	56,02 (11,54)	11,689	<0,001 (9,30; 12,98)	0,97
Com sintomas	236 (38,7%)	44,90 (11,27)			
Não responderam**	02 (0,3%)				
Risco de suicídio atual*					
Sem risco	352 (57,6%)	54,70 (13,01)	7,040	<0,001 (5,22; 9,01)	0,58
Com algum risco	258 (42,2%)	47,67 (10,98)			
Não responderam**	01 (0,2%)				
Sonolência diurna excessiva					
Normal	266 (43,5%)	53,91 (12,78)	3,670	<0,001 (1,91; 5,66)	0,30
Sonolência diurna excessiva	339 (55,5%)	50,13 (12,35)			
Não responderam**	06 (1,0%)				

*Variâncias iguais não assumidas pelo teste de Levene na análise comparativa para médias da variável APD; **Devido ao pequeno tamanho amostrai dos indivíduos que não responderam aos instrumentos de avaliação do sofrimento emocional, a comparação entre grupos se deu apenas entre os grupos com sofrimento emocional e sem sofrimento.

Identificou-se que 52,2% dos estudantes apresentaram sintomas depressivos clinicamente significativos; 73,3% apresentaram sintomas de ansiedade; 38,7% apresentaram sintomatologia compatível com estresse; 42,2% apresentaram algum risco de suicídio atual; e 55,5% apresentaram sintomas de sonolência diurna excessiva.

Análise de regressão linear múltipla demonstrou que, entre os indicadores de sofrimento mental analisados, as variáveis estresse (p <0,001; BCa IC95% = −9,647; −5,182), depressão (p<0,001; BCa IC95% = −7,706; −2,828) e risco de suicídio atual (p = 0,006; BCa IC95% = −4,517; −0,893) associaram-se negativa e significativamente com a APD, sendo que o estresse teve maior competência explicativa (P = −0,286, p < 0,001). Destaca-se que o modelo final assumido foi estatisticamente significativo (F = 17,684, p < 0,001, R^2^ = 0, 267), indicando que o conjunto de variáveis retidas conseguiu explicar 26,7% do desfecho (Tabela 3).

**Tabela 3:** Modelo de regressão linear múltipla para fatores associados à atenção plena disposicional em uma amostra de estudantes universitários da área da saúde, Cuiabá, Mato Grosso, Brasil, 2022

Variáveis	B	Erro padrão	β	BCa IC95%		Valor de *p*	VIF
				Mínimo	Máximo		
Intercepto	48,161	6,7890		34,805	61,517	< 0,001	
Depressão	−5,257	1,120	−0,207	−7,706	−2,828	< 0,001	1,552
Ansiedade	−1,063	1,209	−0,037	−3,405	1,204	0,385	1,347
Estresse	−7,445	1,105	−0,286	−9,647	−5,182	< 0,001	1,418
Risco de suicídio atual	−2,722	0,922	−0,106	−4,517	−0,893	0,006	1,297
Sonolência diurna excessiva	−1,790	0,944	−0,070	−3,714	0,096	0,062	1,058

*Método de inserção: ENTER; R^2^:0,267; F: 17,184 (p < 0,001); Durbin Watson: 1,882. Efeitos dos indicadores de saúde emocional controlados pelas variáveis sociodemográficas e acadêmicas consideradas neste estudo; VIF – Variance Inflation Factor; IC – Intervalo de Confiança.*

## DISCUSSÃO

Esta pesquisa identificou escores moderados de APD e obteve os ISE depressão, estresse e risco de suicídio associados aos níveis de APD em uma amostra de estudantes de cursos das áreas de enfermagem, medicina, nutrição e psicologia. Os escores moderados de APD são concernentes aos obtidos em pesquisas desenvolvidas com populações semelhantes^(26, 27, 28)^.

Em relação às prevalências de ISE aqui descritas, pesquisas reafirmam a vulnerabilidade dessa população em virtude da transição para universidade e dos inúmeros processos adaptativos defrontados com ou sem êxito^(29, 30)^. Pesquisa com 424.128 estudantes universitários brasileiros, em 2018, mostrou o aumento do sofrimento emocional nessa população^(19)^, os quais se agravaram com o longo período pandêmico e sua adjunta complexidade^(29, 30)^.

Logo, esta pesquisa vai ao encontro da literatura ao evidenciar que mais da metade dos estudantes da área da saúde, após recente período de isolamento pandêmico, apresentou sintomas depressivos, sintomas de sonolência diurna excessiva e elevado índice de sintomas de ansiedade. As prevalências obtidas são preocupantes, pois tais indicadores, quando relacionados à APD, predispõem à fraca habilidade de autorregulação, maior vulnerabilidade a mente errante ou em divagação (*mind wandering*), prejuízos à cognição, ruminação de emoções e sentimentos emocionais negativos, inibição das emoções eudemônicas/positivas, precarização da consciência corpórea (habilidades interoceptivas e proprioceptivas), constantes disfunções motivacionais, entre outras^(7, 31, 32, 33, 34)^. Essas condições podem interferir diretamente no rendimento acadêmico, bem como no processo de formação desses estudantes^(9, 33, 35)^.

A APD compreende a capacidade de autorregulação da atenção, de orientação aberta à própria experiência vivenciada^(1, 2)^, capacidade de perceber sensações corporais ou de estar atento ao próprio corpo (interocepção e propriocepção) e a mudança da perspectiva de si^(36)^. No que concerne às vivências emocionais, ainda que sejam alteradas perante as influências sociais e culturais, a sua mais forte natureza é inata e instintiva^(12)^. Tal fato significa que a habilidade de autorregulação é variável, e isso depende diretamente da capacidade de regulação da atenção voluntária^(37)^. Assim, apesar de ser uma questão de nível evolutivo e genético, a APD pode ser cultivada e ampliada singularmente^(2)^, podendo proteger os jovens de preocupações excessivas, ruminação e medo^(38)^.

As associações obtidas neste estudo confirmam que o aumento dos escores de ISE diminui a APD, de forma consoante a achados da literatura prévia. Pesquisas demonstram que a dificuldade de resposta ao estresse, os sintomas de depressão e a presença de ideação suicida exercem uma influência negativa na APD de estudantes da área da saúde^(11, 16)^.

As graduações na área da saúde se diferem das outras formações acadêmicas, pelo seu alto potencial de adoecimento mental, principalmente pela natureza interativa com o sofrimento humano em momentos de fragilidade física e psíquica que tais cursos exigem. Inseguranças e preocupações podem elevar os níveis de estresses dos estudantes, impactando negativamente sua saúde mental, emocional e capacidade de concentração^(8, 10, 11)^.

Nesse sentido, estudo brasileiro realizado com estudantes universitários de enfermagem evidenciou considerável decréscimo da APD diante da insuficiência de horas de sono e presença de sintomatologia de estresse^(26)^.

O estresse, apesar de não ser uma emoção, é uma experiência emocional associada a aspectos bioquímicos, fisiológicos, cognitivos e comportamentais^(23)^, ou, ainda, de múltiplas dimensões, que inclui o interno (psicofisiologia) e o externo (ambiente), além de interações materiais, sociais e culturais vivenciadas em um determinado contexto cotidiano^(2)^. No âmbito da vida contemporânea, incluindo a vivência universitária de estudantes da área da saúde e suas particularidades, a resposta ao estresse persistente sobrecarrega sobremaneira os recursos fisiológicos, humorais e neurais^(8, 16)^, podendo ocasionar o decréscimo da APD. Entre os estudantes e profissionais da área da saúde, o reflexo da diminuição dos níveis de APD gera, entre outros, negligência do cuidado pessoal, falta de atitude autocompassiva, dificuldades de relacionar-se em grupo e ser compassivo com o outro^(39, 40, 41)^.

Outro ISE associado à diminuição da APD foram os sintomas depressivos. Sabe-se da interação complexa da depressão entre fatores sociais, psicológicos e biológicos^(42)^. Eventos da vida, como adversidades, perdas, entre outros, contribuem e podem catalisar o seu desenvolvimento^(42, 43)^. Como um importante preditor, os sintomas depressivos, especialmente entre os jovens universitários diante das adversidades, podem sinalizar diversos agravos à saúde, sobretudo nas funções cognitivas, comportamentais e relacionais estritamente vinculadas à capacidade de APD, afetando diretamente o rendimento acadêmico e outras esferas da vida^(10)^.

A última associação obtida foi o risco de suicídio atual, que contribuiu para o decréscimo da capacidade de APD. Compreende-se o risco de suicídio atual não como uma emoção, mas sim como uma forma avaliativa de um sofrimento emocional profundo que considera todo um processo investigativo do comportamento suicida detectado^(43)^. Estudo realizado com estudantes chineses de cursos de medicina evidenciou que indivíduos em sofrimento que apresentam risco de suicídio podem apresentar dificuldade em lidar com as emoções e apresentar baixa a regulação emocional que, por sua vez, podem levar a um decréscimo agudo ou crônico de habilidades como a APD^(10)^.

Assim sendo, o cultivo da APD por meio da utilização de estratégias corpóreo-mentais baseadas em *mindfulness* ou associadas a abordagens autocompassivas conseguem motivar experiências de consciência e aceitação do momento presente, podendo ajudar os indivíduos a identificar os seus sofrimentos emocionais e ser menos sobrecarregados por sintomas predominantes, como ansiedade, desespero e depressão^(2, 39, 44)^, que afloram em momentos de alta carga de estresse similares aos vividos recentemente com a crise global da COVID-19^(40)^.

Recentemente, pesquisas mostraram que programas de *mindfulness* associados a outras práticas como *yoga* melhoraram significativamente o nível de atenção plena, autocompaixão e compaixão de estudantes de enfermagem^(41)^, bem como exerceram uma influência de proteção do bem-estar de estudantes universitários^(45)^.

Ademais, é importante salientar que, apesar de o indicador emocional de ansiedade não ter sido significativo neste estudo, pesquisas recentes mostram que estudantes da área da saúde podem ter uma redução da APD quando apresentam sintomas de ansiedade (apreensão, preocupação e medo), que são, geralmente, acompanhados de sintomas físicos, como tensão muscular, distúrbios do sono, inquietação, respiração rápida, tremores e fadiga, que podem causar danos à vida pessoal e coletiva tanto dentro quanto fora do ambiente acadêmico^(11, 40, 41)^.

### Limitações do estudo

Diante dos achados, acredita-se que este estudo traz uma importante contribuição científica, porém apresenta algumas limitações a serem descritas, como o tipo de delineamento transversal, que mensura as variáveis simultaneamente, sem distinção estrutural entre variáveis preditoras e de desfecho, permitindo conclusões restritas sobre causalidade ou precedência temporal. Ressalta-se ainda que a amostra por conveniência e o fato de o estudo ter sido realizado em apenas um dos *campi* de uma única instituição de ensino superior pública podem não refletir as características representativas de toda a população. Futuramente, novos estudos poderão abarcar uma base amostral mais ampla e incluir estudantes da área da saúde de diferentes instituições, regiões, estados ou países.

### Contribuições para a área da enfermagem

Os achados podem ampliar o escopo de conhecimento, visando aprimorar o manejo dos cuidados em saúde entre os estudantes universitários da área da saúde. A pesquisa pode ser útil para o planejamento de políticas universitárias e de promoção de saúde mental de estudantes da área da saúde, considerando o potencial salutar da APD na vida das pessoas.

## CONCLUSÕES

A amostra de estudantes da área da saúde deste estudo apontou escores moderados de APD e que o aumento dos ISE de estresse, depressão e risco de suicídio atual se associaram com a diminuição da APD no grupo investigado. Nesse sentido, o diagnóstico situacional promovido pela presente pesquisa pode contribuir para a elaboração de estratégias mais assertivas quanto à promoção da regulação emocional e fortalecimento da APD de estudantes de cursos da área da saúde.

## Data Availability

https://doi.org/10.48331/scielodata.GPEFJP
